# The CPLANE protein Fuzzy regulates ciliogenesis by suppressing actin polymerization at the base of the primary cilium via p190A RhoGAP

**DOI:** 10.1242/dev.202322

**Published:** 2024-03-28

**Authors:** Rhythm Sharma, Rita Kalot, Yossef Levin, Sima Babayeva, Nadezda Kachurina, Chen-Feng Chung, Karen J. Liu, Maxime Bouchard, Elena Torban

**Affiliations:** ^1^ Department of Medicine and Department of Physiology, McGill University, Montreal H4A 3J1, QC, Canada; ^2^ Metabolic Disorders and Complications Program, Research Institute of the McGill University Health Centre, McGill University, Montreal H4A 3J1, QC, Canada; ^3^ Centre for Craniofacial and Regenerative Biology, King's College London, London SE1 9RT, UK; ^4^ Rosalind and Morris Goodman Cancer Institute, Department of Medicine of the McGill University, McGill University, Montreal H3A 1A3, QC, Canada

**Keywords:** Planar cell polarity effectors, CPLANE, Primary cilium, Ciliogenesis, Actin, Cytoskeleton, ARHRhoGAP35, Mouse

## Abstract

The primary cilium decorates most eukaryotic cells and regulates tissue morphogenesis and maintenance. Structural or functional defects of primary cilium result in ciliopathies, congenital human disorders affecting multiple organs. Pathogenic variants in the ciliogenesis and planar cell polarity effectors (CPLANE) genes *FUZZY*, *INTU* and *WDPCP* disturb ciliogenesis, causing severe ciliopathies in humans and mice. Here, we show that the loss of Fuzzy in mice results in defects of primary cilia, accompanied by increased RhoA activity and excessive actin polymerization at the basal body. We discovered that, mechanistically, Fuzzy interacts with and recruits the negative actin regulator ARHGAP35 (also known as p190A RhoGAP) to the basal body. We identified genetic interactions between the two genes and found that a mutant *ArhGAP35* allele increases the severity of phenotypic defects observed in *Fuzzy*^−/−^ mice. Based on our findings, we propose that Fuzzy regulates ciliogenesis by recruiting ARHGAP35 to the basal body, where the latter likely restricts actin polymerization and modifies the actin network. Our study identifies a mechanism whereby CPLANE proteins control both actin polymerization and primary cilium formation.

## Teaser

**Summary:** Examination of mutant cells as well as multiple single and double knockout mice reveals a mechanism whereby the CPLANE protein Fuzzy controls actin polymerization and ciliogenesis via p190ARhoGAP.

## INTRODUCTION

The primary cilium is an evolutionarily conserved, microtubule-based organelle that protrudes from the apical surface of most eukaryotic cells and functions as a signaling nexus to coordinate tissue morphogenesis (Anvarian et al., 2019). Structural or functional defects in cilia often manifest as multi-organ diseases termed ciliopathies. These include Bardet–Biedl syndrome, nephronophthisis, Joubert syndrome, and multiple other disorders (reviewed by Hildebrandt et al., 2011). Ciliopathies are associated with cilia defects and manifest in anatomic aberrations, such as cystic kidneys, obesity, craniofacial abnormalities, neural tube and skeletal defects, and many others (Hildebrandt et al., 2011).

Mutations in over 187 genes have been implicated in the 35 established ciliopathies. Some ciliopathy-associated gene mutations affect intrinsic ciliary proteins, whereas others influence non-ciliary proteins that are nevertheless crucial for cilial assembly and function (Reiter and Leroux, 2017). Among the mutant genes associated with ciliopathies are the genes encoding ciliogenesis and planar cell polarity effector (CPLANE) proteins. Mutations in human CPLANE genes cause the embryonically lethal short rib polydactyly syndrome, as well as Joubert syndrome, orofacial-digital syndrome, Bardet–Biedl syndrome and nephronophthisis (Martín-Salazar and Valverde, 2022; Zhang et al., 2018; Gray et al., 2009; Toriyama et al., 2016; Barrell et al., 2022). CPLANE proteins interact with ciliogenic proteins, such as Jbts17 (Cplane1), Rsg1 (Cplane2) and Rab23 (Toriyama et al., 2016; Gerondopoulos et al., 2019; Agbu et al., 2018; Brooks and Wallingford, 2013). They were also found to recruit several ciliary regulators to the basal body (Toriyama et al., 2016; Zilber et al., 2013). Mutations of CPLANE genes in mice or their knockdown in *Xenopus laevis* result in defective ciliation, whereby cilia are lost or shortened, and lead to a wide range of complex abnormalities (Gray et al., 2009; Barrell et al., 2022; Park et al., 2006; Heydeck et al., 2009; Seo et al., 2011; Heydeck and Liu, 2011).

CPLANE genes are evolutionarily conserved and were first identified in *Drosophila melanogaster* where they are known as planar cell polarity (PCP) effectors that participate in downstream PCP pathways (Maung and Jenny, 2011; Gubb and Garcia-Bellido, 1982; Collier and Gubb, 1997; Adler et al., 1994). In *Drosophila*, PCP signaling regulates cell polarity within the tissue plane, thereby determining the uniform orientation of actin-rich hairs (trichomes) on an insect's wing cells (Gubb and Garcia-Bellido, 1982). The PCP effectors Fuzzy (Fuz in mouse), Inturned (Intu in mouse) and Fritz (WDPCP in vertebrates) constrain the number and directionality of trichomes possibly by suppressing actin polymerization on the proximal side of the wing cell where they localize. Mutations of PCP effectors result in dysregulation of actin polymerization, evident in the formation of multiple, randomly oriented hairs on each cell. Thus, PCP effectors likely act as negative regulators of actin polymerization in fly wing cells.

Recent evidence points to the importance of actin regulation in ciliogenesis (Copeland, 2020; Kim et al., 2010; Hoffman and Prekeris, 2022). Both the initiation and maintenance of primary cilia require finely tuned actin; excessive actin polymerization negatively affects the formation of primary cilia (Kim et al., 2010; Boisvieux-Ulrich et al., 1990; Yan and Zhu, 2013; Antoniades et al., 2014). Actin polymerization at the basal body is in part controlled by the RhoA GTPase/ROCK pathway (Copeland, 2020). One of the major mammalian regulators of RhoA GTPases is a GTPase-activating protein ARHGAP35 (also known as p190A RhoGAP, hereafter termed p190A) (Heraud et al., 2019); it is involved in cell motility, proliferation, and neuronal morphogenesis (Heraud et al., 2019). Recently, it was also shown to be crucial for ciliogenesis (Stewart et al., 2016; Streets et al., 2020). For example, loss of functional p190A at the basal body of mouse renal tubular cells results in a considerable decrease in ciliary length, accompanied by glomerulocystic transformation of the proximal nephron (Stewart et al., 2016). Furthermore, the loss of p190A in mouse embryonic fibroblasts (MEFs) prevents cilia elongation through inappropriate activation of Rho/ROCK signaling (Stewart et al., 2016).

In light of their function in *Drosophila* as actin regulators, their impact on cilial structure in mouse and *Xenopus* models, and the emerging role of actin regulation in primary cilium formation, we hypothesized that CPLANE proteins might regulate ciliogenesis, in part, by controlling the actin cytoskeletal network at the basal body. In this study, we show that the knockout of *Fuzzy* in mice leads to glomerulocystic kidney phenotype and ciliary defects accompanied by excessive actin polymerization at the basal body. We demonstrate that inhibition of actin polymerization rescues ciliation in *Fuzzy* mutant MEFs and kidney explants *ex vivo*. We show that *Fuzzy* genetically and biochemically interacts with p190A and controls its localization at the ciliary base. Thus, our data suggest that Fuzzy recruits p190A to the cilium, where it likely inhibits excessive actin polymerization and allows proper ciliogenesis. Our results provide a plausible mechanism that links the impact of the CPLANE protein Fuzzy on actin regulation with its role in primary cilium formation.

## RESULTS

### Glomerulocystic kidney phenotype and deficient ciliogenesis in E16.5 *Fuzzy*^−/−^ mouse kidneys

**Fig. 1.**
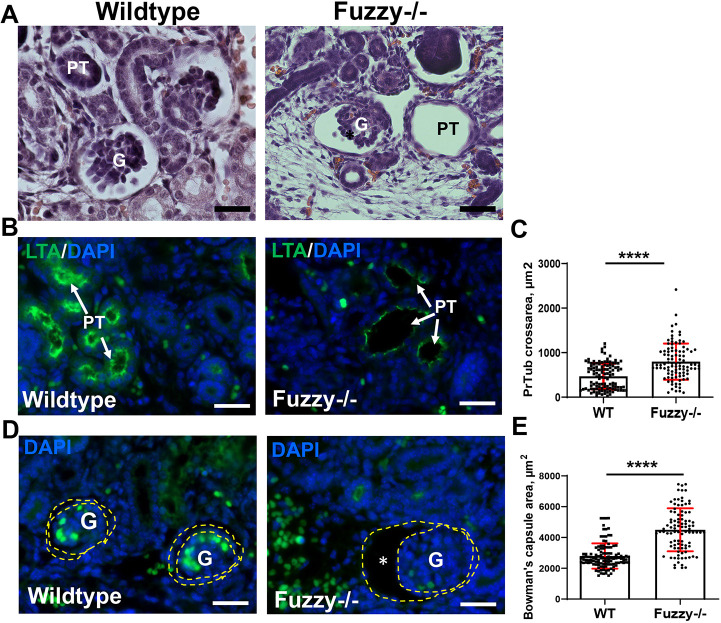
**Tubular and glomerular cysts in E16.5 *Fuzzy^−/−^* kidneys.** (A) Hematoxylin and Eosin staining of E16.5 wild-type and *Fuzzy^−/−^* kidneys. Note cystic transformation of proximal tubules (PT) and glomeruli (G) in *Fuzzy^−/−^* kidneys. (B) Representative fluorescence images of E16.5 wild-type and *Fuzzy^−/−^* sections stained with LTA. White arrows point to proximal tubules visualized with LTA (green staining at the predominantly luminal side of the tubules). (C) Statistical analysis of proximal tubule (PrTub) cross-sectional area. *n*=120 (WT) and *n*=112 (*Fuzzy^−/−^*) structures were analyzed. (D) Representative fluorescence images of E16.5 wild-type and *Fuzzy^−/−^* kidney sections with the LTA staining of proximal tubules, where visible. White arrows point at glomeruli, asterisk marks cystic Bowman's capsule. Note that blood cell autofluorescence is seen as green staining. (E) Statistical analysis of glomerular cross-sectional area. *n*=137 (WT) and *n*=111 (*Fuzzy^−/^*^−^) glomeruli were measured in three E16.5 embryos per genotype. ****P<0.0001 (unpaired two-tailed *t*-test).

**Fig. 2.**
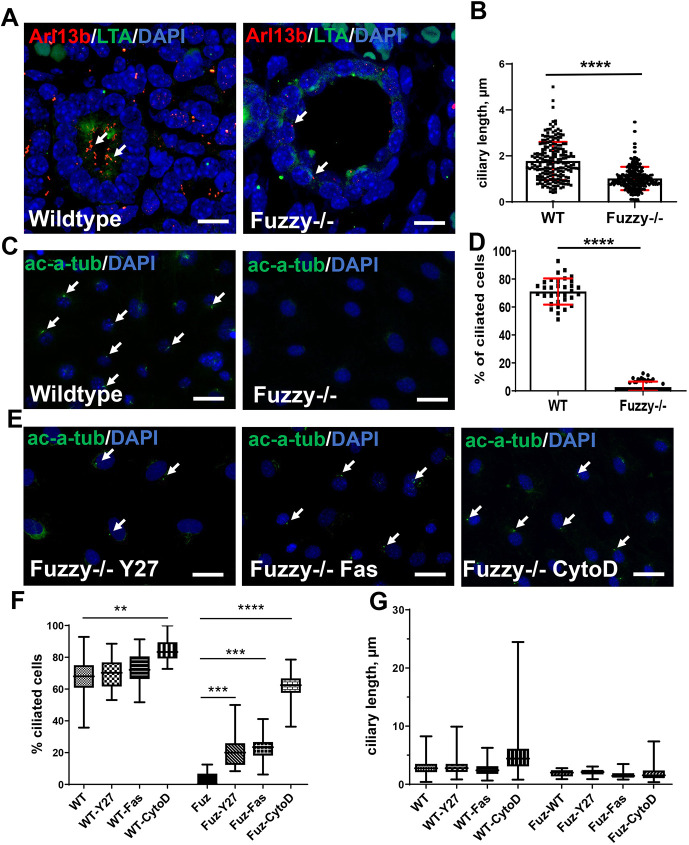
**Impaired ciliogenesis in *Fuzzy^−/−^* cells is rescued *in vitro* by inhibiting actin polymerization and RhoA kinase.** (A) Representative confocal images of cilia in proximal tubules of E16.5 kidneys. Primary cilia were visualized with anti-Arl13b antibody (red), proximal tubules were identified with LTA (green) and nuclei were stained with DAPI (blue). (B) Analysis of ciliary length in the cells of proximal tubules *in vivo*. *n*=201 (WT) and *n*=321 (*Fuzzy^−/−^*). (C) Ciliogenesis in wild-type and *Fuzzy^−/−^* MEFs. White arrows point to primary cilia, which were visualized with anti-acetylated-α-tubulin antibody (green); nuclei were stained with DAPI (blue). (D) Percentage of ciliated MEFs. Ten images per experiment per genotype were analyzed; three independent experiments were carried out. (E) Representative fluorescence images of *Fuzzy^−/−^* MEFs treated with inhibitors of ROCK (50 µM Y27632 and 1 µM fasudil) and of actin polymerization (0.5 µM cytochalasin D). Cilia were detected with anti-acetylated-α-tubulin antibody (green); nuclei were stained with DAPI (blue). (F) Percentage of ciliated cells (ciliation) in *Fuzzy^−/−^* MEFs treated with indicated inhibitors as in E. (G) Statistical analysis of ciliary length. *n*=407 (WT), *n*=271 (WT+Y27632), *n*=249 (WT+fasudil), *n*=260 (WT+CytoD), *n*=7 (*Fuzzy^−/−^*), *n*=35 (*Fuzzy^−/−^*+Y27632), *n*=37 (*Fuzzy^−/−^*+fasudil), *n*=201 (*Fuzzy^−/−^+*CytoD) cilia were measured. *P≤0.05, **P≤0.01, ***P≤0.001, ****P<0.0001 (one way ANOVA was used to ascertain the differences between a non-treated and all treated groups followed by an unpaired two-tailed *t*-test to compare the differences between the untreated and each treated group). The upper and lower limits of the boxes in F and G represent the first quartile (lower end) and the third quartile (upper end); whiskers represent minimum to maximum data points, and the horizontal line represents the median. Scale bars: 10 µm (A); 20 µm (C,E).

The majority of *Fuzzy^−/−^* mice in our colony die *in utero* at embryonic day (E) 14.5 with few surviving until E16.5. To assess this later developmental stage, we analyzed E16.5 kidneys and noted profound dilatation of both proximal tubules and juxtamedullary glomeruli (Fig. 1A-C, Figs S1, S2), resembling the phenotype of glomerulocystic disease in humans (reviewed by Bissler et al., 2010). The glomeruli in *Fuzzy^−/−^* mice appeared to develop properly, based on the number and arrangement of glomerular epithelial cells (podocytes) and size of the glomerular tuft in control and mutant animals (Wang et al., 2021) (Fig. S2). However, the Bowman's space was significantly dilated (Fig. 1D,E). Although the mechanisms of glomerulocystic disease are not well understood, it has been linked to abnormal ciliary function (Watnick and Germino, 2003). We and others have previously reported abnormal cilia formation in *Fuzzy^−/−^* mice (Gray et al., 2009; Heydeck et al., 2009; Wang et al., 2021). Indeed, the primary cilia in the E16.5 renal tubules were significantly shorter (Fig. 2A,B); *Fuzzy^−/−^* MEFs had very few primary cilia (Fig. 2C,D).

### Inhibitors of ROCK and actin polymerization rescue ciliogenesis in *Fuzzy^−/−^* cells *in vitro* and *ex vivo*

Given the role of Fuzzy in both regulation of actin cytoskeleton in *Drosophila* and cilia formation in vertebrates, we explored the hypothesis that Fuzzy might regulate ciliogenesis directly through its effects on actin polymerization. First, we treated wild-type and the largely unciliated *Fuzzy^−/−^* MEFs with 50 µM Y27632 or 0.5 µM fasudil, both inhibitors of RhoA kinases (ROCKs). Whereas the percentage of ciliated cells (ciliation) in wild-type MEFs did not increase significantly, treatment of *Fuzzy^−/−^* MEFs with either ROCK inhibitor resulted in an approximately ninefold increase in ciliation (from ∼2.5% to ∼22%) (Fig. 2E,F). Ciliary length was not significantly affected in MEFs of either genotype (Fig. 2G). Similarly, treatment with 0.5 µM cytochalasin D, a strong inhibitor of actin polymerization, resulted in only a mild increase in the percentage of ciliated wild-type MEFs, whereas the percentage of ciliated *Fuzzy^−/−^* MEFs increased dramatically (about 24-fold) from ∼2.5% to ∼60% (Fig. 2E,F). Ciliary length increased in both *Fuzzy^+/+^* and *Fuzzy^−/−^* MEFs (Fig. 2G). Of note, we reported no differences in the length of F-actin stress fibers in *Fuzzy^−/−^* MEFs compared with wild-type cells (Fig. S3A,B).

**Fig. 3.**
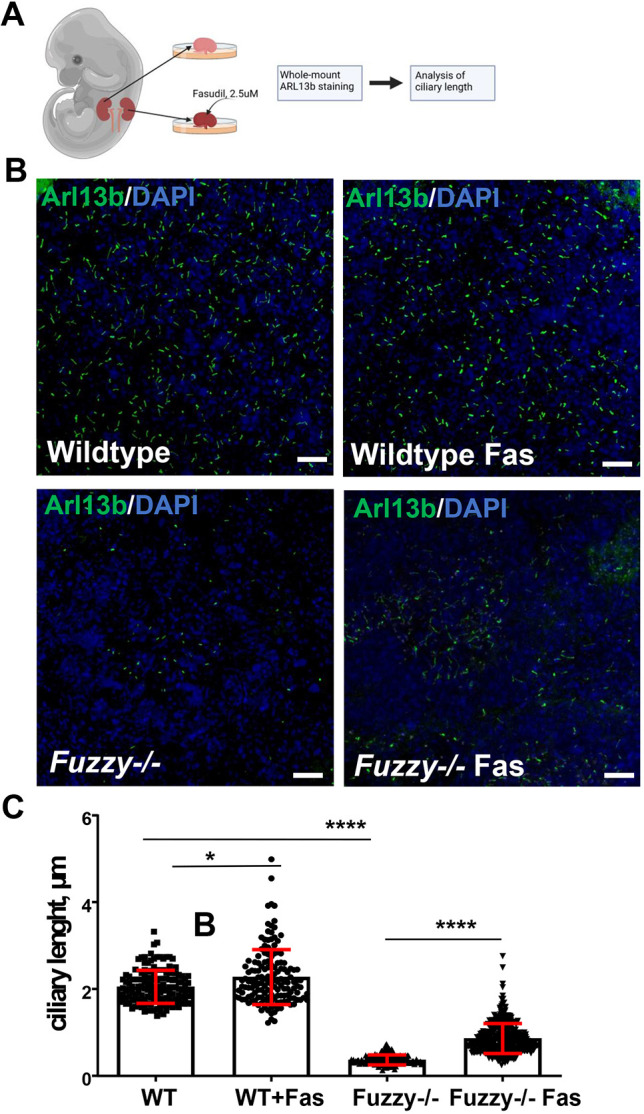
**Effect of fasudil on ciliogenesis in *Fuzzy^−/−^* embryonic kidneys *ex vivo*.** (A) Schematic of kidney explant experiments. Note that one kidney from the same embryo is treated and the other is used as internal control (created with BioRender.com). (B) Representative confocal images of whole-mount staining with anti-Arl13b antibody (green) and DAPI (blue) of kidney explants after 3 days incubation with or without 2.5 µM fasudil. Scale bars: 25 µm. (C) Statistical analysis of ciliary length. Three kidneys per condition were examined in two independent experiments; *n*=450 (WT), *n*=320 (WT+Fas), *n*=390 (*Fuzzy^−/−^*), *n*=622 (*Fuzzy^−/−^*+Fas) cilia were measured. *P≤0.05, ****P<0.0001 (unpaired two-tailed *t*-test).

*Ex vivo* embryonic kidney explants have been widely used as an informative model to identify cellular processes and molecules that impact kidney development (Grobstein, 1953; Herzlinger et al., 1994). Treatment of wild-type kidney explants for 3 days with 2.5 µM fasudil (Fig. 3A) increased ciliary length only slightly (about ∼9%) from a mean of 1.95 µm to 2.12 µm (Fig. 3B,C). By contrast, the length of stunted cilia in *Fuzzy^−/−^* kidney explants increased by ∼209% from 0.44 µm to 0.92 µm (Fig. 3C), indicating a selective effect of fasudil on ciliogenesis in mutant *Fuzzy^−/−^* but not in wild-type kidney explants.

### RhoA activity and actin polymerization in *Fuzzy*^−/−^ MEFs and tissues

**Fig. 4.**
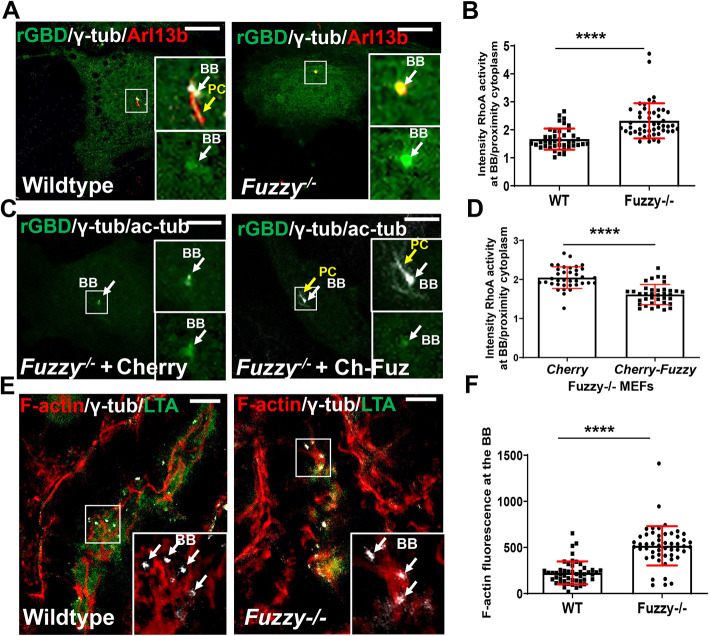
**RhoA activity and actin polymerization in *Fuzzy^−/−^* MEFs and tissues.** (A) Representative fluorescence images of wild-type and *Fuzzy^−/−^* MEFs expressing the GFP-rGBD reporter of RhoA activity: primary cilia (PC) were detected with anti-Arl13b antibody (red), basal bodies (BB) were visualized with anti-γ-tubulin antibody (white), and GFP-rGBD is visualized as green. (B) Statistical analysis of fluorescence intensity of GFP-rGBD at the basal body; *n*=41 (WT) and *n*=49 *(Fuzzy^−/−^*) cells were analyzed. (C) Representative fluorescence images of *Fuzzy^−/−^* MEFs transfected with either Cherry plus rGFP-GBD or Cherry-Fuzzy plus GFP-rGBD. Basal body (white arrows) and primary cilia (yellow arrows) were visualized with anti-γ-tubulin and anti-acetylated-α-tubulin (both white), Cherry (red to identify transfected cells; not shown) and GFP-rGBD (green). (D) Statistical analysis of GFP-rGBD fluorescence intensity at the basal body; *n*=37 (Cherry), *n*=37 (Fuzzy-Cherry). (E) Representative fluorescence images of F-actin (detected with phalloidin staining; red) at the basal body visualized with anti-γ-tubulin antibody (white) in the E14.5 embryonic kidney proximal tubule identified with LTA (green). (F) Statistical analysis of F-actin fluorescence intensity at the basal body in wild-type and *Fuzzy* ^−/−^ tubule cells; *n*=128 (WT) and *n*=112 (*Fuzzy^−/−^*) cells from 4 embryos per genotype were analyzed. ****P<0.0001 (unpaired two-tailed *t*-test). Insets in A,C,E show 2.5× magnifications of the boxed areas. Scale bars: 5 µm (A,C); 20 µm (E).

Given that our results could be explained by excessive RhoA signaling in *Fuzzy^−/−^* cells, we first measured RhoA activity by a GST-Rhotekin pull-down assay in whole-cell lysates of wild-type and *Fuzzy^−/−^* MEFs; however, no differences were detected (Fig. S4A). Of note, no differences were detected in the activity of Rac1 or Cdc24 (cell division cycle 42; HGNC1736) (Fig. S4B,C), although Rac1 activity somewhat decreased but it did not reach significance (Fig. S4B). We then used a Rhotekin-GFP reporter (GFP-rGBD) to assess spatial differences in RhoA activity in the cells (Pertz and Hahn, 2004). We detected the fluorescence reporter signal at the basal bodies (visualized with anti-γ-tubulin antibody) in both ciliated wild-type cells and *Fuzzy^−/−^* mutant cells that lack cilia (Fig. 4A,B, Fig. S5A,B). However, the average fluorescence intensity of the RhoA reporter at the basal body normalized for the intensity in the proximal cytoplasm (to account for transfection efficiency of RhoA-rGBD sensor; Fig. S5A) was significantly higher in *Fuzzy^−/−^* fibroblasts than in wild-type cells (Fig. 4B). These results signify a profound increase in RhoA activity at the basal body in the absence of functional Fuzzy protein.

To affirm that elevated RhoA activity in the *Fuzzy^−/−^* MEFs was due to the loss of Fuzzy function, we performed rescue experiments, in which mutant MEFs were transfected with RhoA sensor in combination with either Cherry or Cherry-Fuzzy expression constructs (Fig. 4C,D). We previously established that transfection of Fuzzy cDNA into *Fuzzy^−/−^* MEFs rescues ciliogenesis (Seo et al., 2011). Indeed, re-establishment of primary cilium was confirmed in the cells transfected with Cherry-Fuzzy, but not in those transfected with Cherry (Fig. 4C, Fig. S5B). The fluorescence intensity of the RhoA sensor in the Cherry-positive cells was measured as described for Fig. 4A,B. The average fluorescence intensity of GFP-rGBD in the Cherry-Fuzzy-transfected cells (normalized for GFP-rGBD intensity in the cytoplasm proximal to basal body, as shown in Fig. S5A) was significantly reduced compared with the cells transfected with Cherry (Fig. 4D, Fig. S5B). This proves that elevated RhoA activity in the *Fuzzy^−/−^* MEFs can be attributed to the loss of functional Fuzzy protein. In the embryonic proximal renal tubular cells of E14.5 mouse wild-type and *Fuzzy^−/−^* embryos (visualized with the marker *Lotus tetragonolobus* agglutinin, LTA) polymerized filamentous actin at the basal body (detected with phalloidin and anti-γ-tubulin antibody, respectively) was ∼60% higher in *Fuzzy^−/−^* compared with control tissues (Fig. 4E,F, Fig. S5C).

### Localization of p190A in *Fuzzy*^−/−^ MEFs

**Fig. 5.**
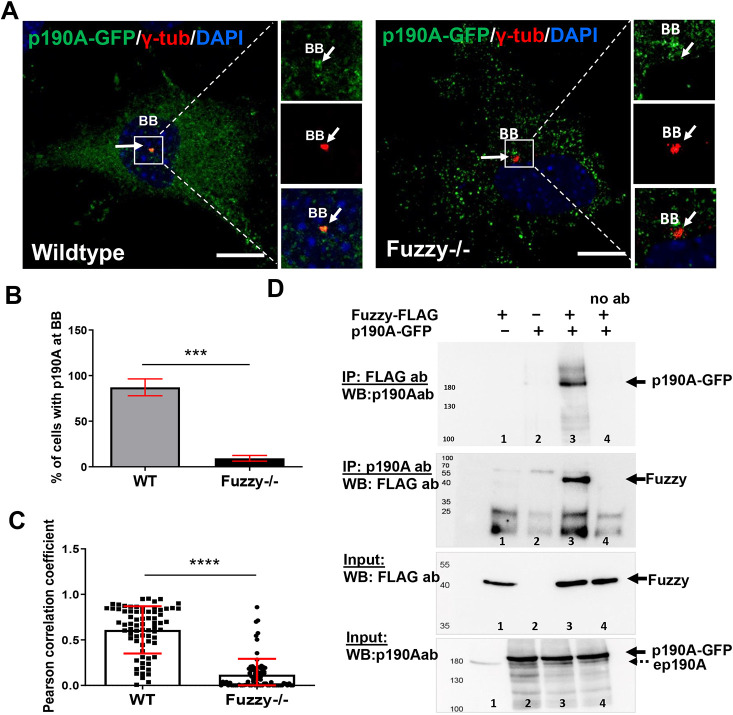
**Interactions between Fuzzy and p190A RhoGAP proteins *in vitro*.** (A) Confocal images of wild-type and *Fuzzy^−/−^* MEFs expressing GFP-p190A at the basal body (BB) visualized with anti-γ-tubulin antibody (red). Scale bars: 5 µm. Boxed areas are shown as separate channels at 2.5× magnification on the right. (B) Percentage of wild-type and *Fuzzy^−/−^* MEFs with GFP-p190A at the basal body (binary analysis ‘yes’ or ‘no’). (C) Pearson correlation coefficient between GFP-p190A and basal body marker; *n*=73 (WT) and *n*=82 (*Fuzzy^−/−^*) cells were analyzed in 3 independent experiments. (D) Co-immunoprecipitation between human Fuzzy-FLAG and human GFP-p190A: the two top panels are co-immunoprecipitations with anti-p190A and anti-FLAG antibodies; the two bottom panels are inputs to detect Fuzzy-FLAG and p190A RhoGFP with respective antibodies. Note that the ep190 band, which represents endogenous p190ARhoGAP, is lower than the transfected GFP-p190A protein band. Lane 1, Fuzzy-FLAG plus GFP-p190A; lane 2, Fuzzy-FLAG; lane 3, GFP-p190A; lane 4, Fuzzy-FLAG plus GFP-p190A. no ab, no antibody was used for co-immunoprecipitation. ***P≤0.001, ****P<0.0001 (unpaired two-tailed *t*-test).

Increased RhoA activity at the basal body may be caused by a lack of proper inhibition of RhoA activity (Copeland, 2020). Indeed, depletion of several mammalian GAPs, including p190A, was recently shown to affect ciliary length (Streets et al., 2020). p190A is localized at the basal body and expresses a strong hydrolyzing activity specifically toward the GTPase RhoA (Stewart et al., 2016). Intriguingly, the inactivating Leu1396 mutation of *ArhGAP35* in mice abolishes GAP activity and causes increased actin polymerization at the basal body of renal tubular cells (as a result of loss of p190A protein at the basal body), and glomerulocystic kidney disease (Stewart et al., 2016) with features strikingly similar to the abnormalities found in *Fuzzy^−/−^* mouse kidney (Fig. 1A). Thus, we analyzed p190A localization at the base of the cilium in the *Fuzzy^−/−^* MEFs. Wild-type and *Fuzzy^−/−^* MEFs were transfected with GFP-p190A expression vector, and a GFP fluorescence signal at the basal body (visualized using a γ-tubulin antibody) was identified by confocal microscopy (Fig. 5A). We detected GFP-p190A at the basal body in over 80% of the wild-type cells, whereas only approximately 15% of the *Fuzzy^−/−^* MEFs had a detectable GFP signal (Fig. 5B). Pearson correlation analysis between the GFP signal and the basal body marker indicated a significant decrease in the localization of GFP-p190A at the basal body in mutant cells (Fig. 5C). Of note, Fuzzy protein localization at the basal body of wild-type and *ArhGAP35^D34/D34^* mutant MEFs was indistinguishable (Fig. S6). Importantly, in the absence of Fuzzy, not only was p190A missing at the basal body, but its overexpression did not rescue ciliogenesis (Fig. S7).

### Biochemical interactions between Fuzzy and p190A

We detected interactions between Fuzzy-FLAG and GFP-p190A by reciprocal co-immunoprecipitation assays using either anti-FLAG or anti-p190A antibodies to detect GFP-p190A and Fuzzy-FLAG, respectively (Fig. 5D). Importantly, we can conclude that the Fuzzy-p190A interaction is specific because p190A does not bind to Inturned, another CPLANE protein that is known to act together with Fuzzy (Fig. S8).

### Genetic interactions between *Fuzzy* and *ArhGAP35*

**Fig. 6.**
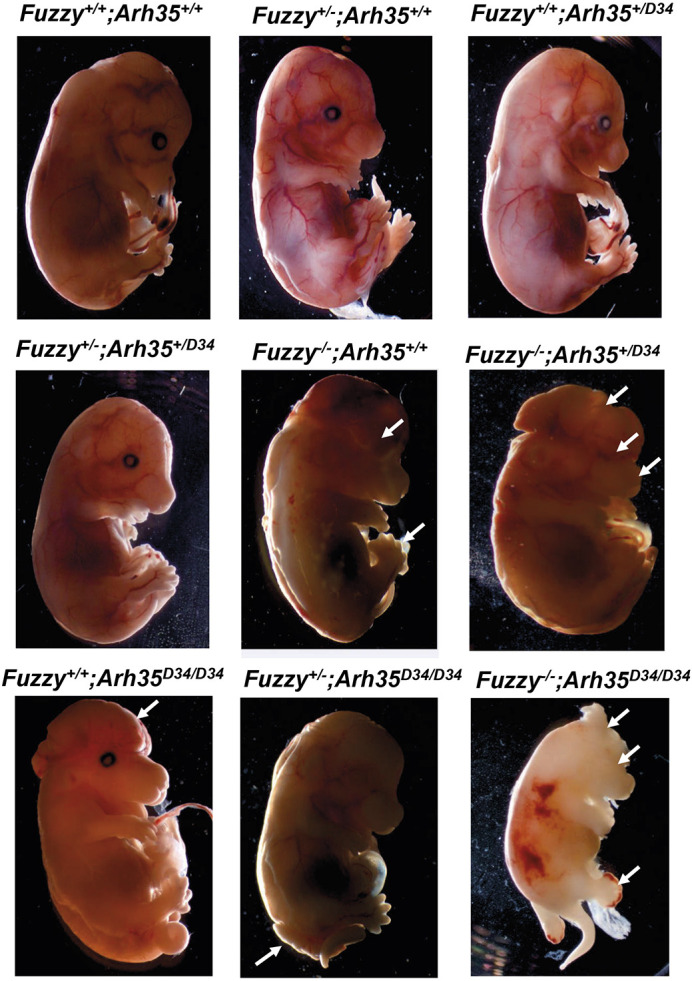
**Interactions between *Fuzzy* and *ArhGAP35* genes *in vivo*.** Representative images of E14.5 embryos from various crosses; genotypes are indicated above of each image. White arrows point to specific malformations, such as exencephaly, spina bifida, microphthalmia, craniofacial defects and polydactyly. See Table 1 for further details of the incidence of the various phenotypes.

The biochemical and functional links between Fuzzy and p190A prompted us to investigate potential genetic interactions between the two genes. *Fuzzy^+/−^* mice were crossed with *ArhGAP35^+/D34^* mutants (to obtain compound heterozygous *Fuzzy^+/−^;ArhGAP35^+/D34^* animals) and studied after weaning. The compound heterozygotes were viable and indistinguishable from wild-type or single heterozygotes. No albuminuria was detected in double heterozygotes at 8 months (Fig. S9). However, the genotype distribution among weaned animals was somewhat skewed toward wild-type and single heterozygotes: 30% (wild type), 30% (*Fuzzy^+/^*^−^), 22% (*ArhGAP^+/D34^*), 17.9% (*Fuzzy^+/−^;ArhGAP35^+/D34^*) (Table S1), suggesting possible attrition of some double heterozygous animals during gestation. Indeed, we noted that 3.7% of the compound heterozygous E14.5 embryos had disturbances of normal development, including 2.5% with exencephaly (embryonically lethal phenotype) (Table 1). Crosses between the double heterozygous animals were undertaken to further reduce the frequency of wild-type alleles. All animals were collected at E14.5 (because the majority of *Fuzzy^−/−^* mice do not survive past this stage) and examined for neural tube defects, microphthalmia, polydactyly and developmental delay, the phenotypes reported in *Fuzzy* or *ArhGAP35^D34/D34^* homozygotes. The embryos with three mutant alleles (either *Fuzzy^−/−^;ArhGAP35^+/D34^* or *Fuzzy^+/−^;ArhGAP35^D34/D34^*) displayed a range of phenotypes resembling those in homozygous *Fuzzy^−/−^* or *ArhGAP35^D34/D34^* animals. However, the frequencies of severe phenotypes were much higher in mutants with a loss of three alleles. Furthermore, we detected additional phenotypes not seen in the single homozygotes (Fig. 6, Table 1). For example, among 14 *Fuzzy^−/−^* homozygotes, we detected no neural tube defect phenotype; however, the loss of one *ArhGAP35* mutant allele on *Fuzzy^−/−^* background resulted in 60% exencephalic embryos. Likewise, among 12 *ArhGAP35^D34/D34^* embryos, we did not detect any animals with microphthalmia; however, removal of one *Fuzzy* allele on the *ArhGAP35^D34/D34^* background resulted in 20% microphthalmia in the *Fuzzy^+/−^;ArhGAP35^D34/D34^* animals. All three E14.5 double-homozygous *Fuzzy^−/−^;ArhGAP35^D34/D34^* animals exhibited severe developmental delay, 100% cranial neural tube defects, polydactyly and microphthalmia (Fig. 6, Table 1).

**Table 1.**
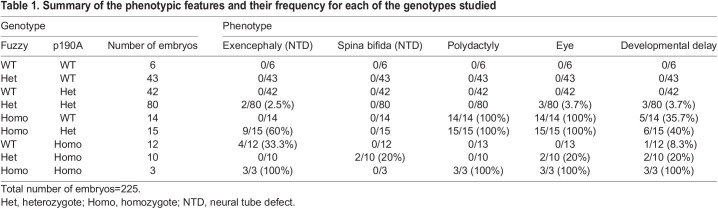
Summary of the phenotypic features and their frequency for each of the genotypes studied

**Fig. 7.**
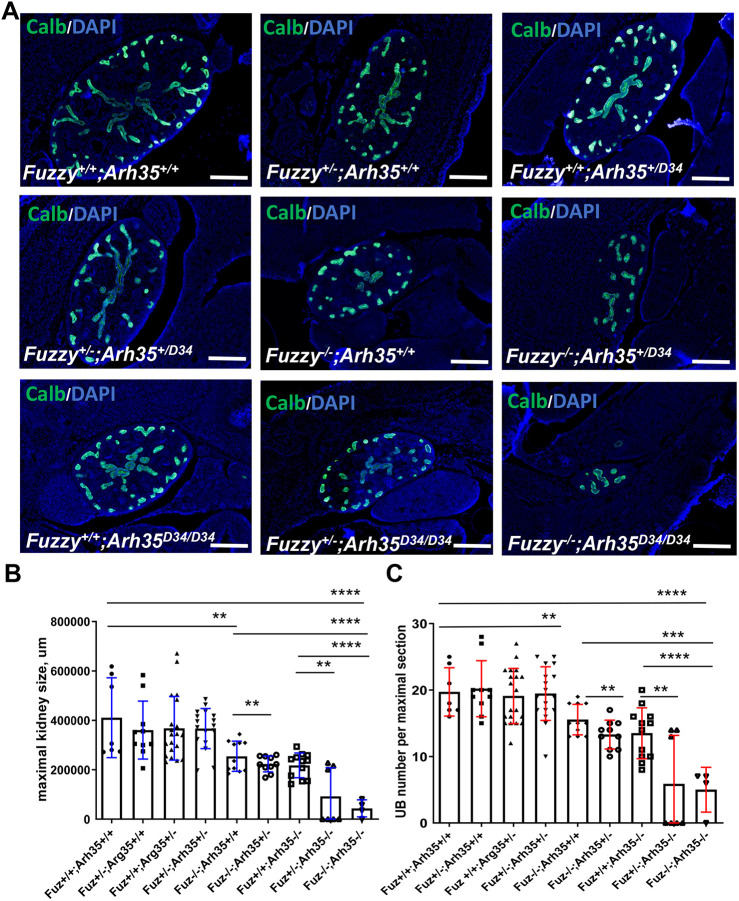
**Interactions between *Fuzzy* and *ArhGAP35* genes during kidney morphogenesis *in vivo*.** (A) Representative images of maximal E14.5 kidney cross-sections from each of the nine genotypes. Branching UBs were visualized with anti-calbindin antibody (green); nuclei were stained with DAPI (blue). Scale bars: 200 µm. (B) Statistical analysis of the maximal section size. The size of two maximal sections per kidney per embryo was measured; each dot represents individual measurement. Note that for each embryo lacking kidneys, one ‘0’ is assigned to calculate the average size. (C) Statistical analysis of UB number in the kidney maximal sections for each genotype. One ‘0’ is assigned for each embryo lacking kidneys. **P≤0.01, ***P≤0.001, ****P<0.0001 (one-way ANOVA was used to compare the wild-type group with the embryos of all other genotypes, followed by an unpaired two-tailed *t*-test to compare each group).

We previously reported that E14.5 *Fuzzy^−/−^* embryos display renal hypoplasia due to deficient branching of the ureteric bud (UB) (Wang et al., 2021). We used this informative quantitative trait to analyze genetic interactions between *Fuzzy* and *ArhGAP35*. Paraffin-embedded embryos were sectioned to obtain maximal kidney sections (visible UB structures at the center of the kidneys). We did not detect significant differences among the wild type, single and double heterozygotes (Fig. 7A,B, and not shown). As reported previously (Stewart et al., 2016; Wang et al., 2021), kidneys from both *Fuzzy^−/−^* and *ArhGAP35^D34/D3^* E14.5 embryos were hypoplastic with a significantly reduced number of the UB tips compared with wild-type mice. Additional decrease of wild-type alleles further reduced the number of the UB tips and kidney size (Fig. 7B,C). Importantly, we detected 60% (4/6) anephric animals in the *Fuzzy^+/−^;ArhGAP35^D34/D34^* crosses; the remaining 40% of *Fuzzy^+/−^;ArhGAP35^D34/D34^* animals displayed kidney sizes similar to that of *ArhGAP35^D34/D34^* animals, suggesting a variability of renal phenotypes. In addition, 33% (1/3) of the compound *Fuzzy^−/−^;ArhGAP35^D34/D34^* E14.5 embryos displayed anephria; the remaining 67% of embryos had only tiny dysplastic kidney rudiments that were markedly smaller than in homozygous mutants of either genotype.

**Fig. 8.**
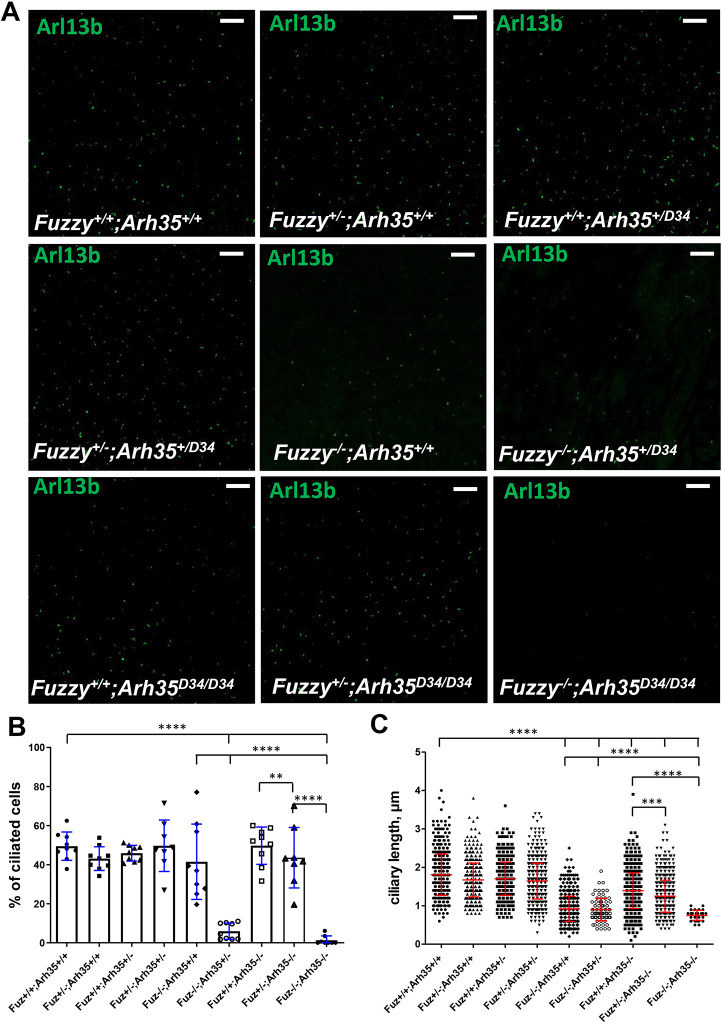
**Interactions between *Fuzzy* and *ArhGAP35* genes control the integrity of primary cilia *in vivo*.** (A) Representative images of maximal E14.5 kidney cross-sections from each of the nine genotypes from *Fuzzy^+/−^;ArhGAP35^+/D34^* crosses. Cilia were visualized with anti-Arl13b antibody (green). Scale bars: 25 µm. (B) Statistical analysis of ciliary length. *n*=610 (*Fuzzy^+/+^;Arh35^+/D34^*), *n*=505 (*Fuzzy^+/−^;Arh35^+/+^*), *n*=695 (*Fuzzy^+/+^;Arh35^+/D34^*), *n*=766 (*Fuzzy^+/−^;Arh35^+/D34^*), *n*=778 (*Fuzzy^−/−^;Arh35^+/+^*); *n*=82 (*Fuzzy^−/−^;Arh35^+/D34^*), *n*=956 (*Fuzzy^+/+^,Arh35^D34/D34^*), *n*=761 (*Fuzzy^+/−^;Arh35^D34/D34^*), *n*=18 (*Fuzzy^−/−^;Arh35^D34/D34^*). (C) Statistical analysis of the percentage of ciliated cells. Seven or eight images for each genotype were taken. The percentage of ciliated cells to the total number of cells (identified with DAPI staining in the image; not shown) was calculated and ciliary length measured. Sections of two embryos per genotype were analyzed. **P≤0.01, ***P≤0.001, ****P<0.0001 (one-way ANOVA was used to compare the wild-type group with the embryos of all other genotypes, followed by an unpaired two-tailed *t*-test to compare each group.

The percentage of ciliated cells and ciliary length were measured in the embryos of all crosses (Fig. 8). Reduced ciliary length was previously reported in both *Fuzzy^−/−^* and *ArhGAP35^D34/D34^* embryos (Gray et al., 2009; Stewart et al., 2016). However, we detected a further decrease in the ciliary length and in the percentage of ciliated cells in the animals with loss of three or four alleles, especially in the *Fuzzy^−/−^*;*ArhGAP3^+/D34^* genotype. Only a few cells were ciliated in the double-homozygous embryos (Fig. 8).

### The ROCK inhibitor fasudil rescues *Fuzzy*^−/−^ renal hypoplasia *ex vivo*

**Fig. 9.**
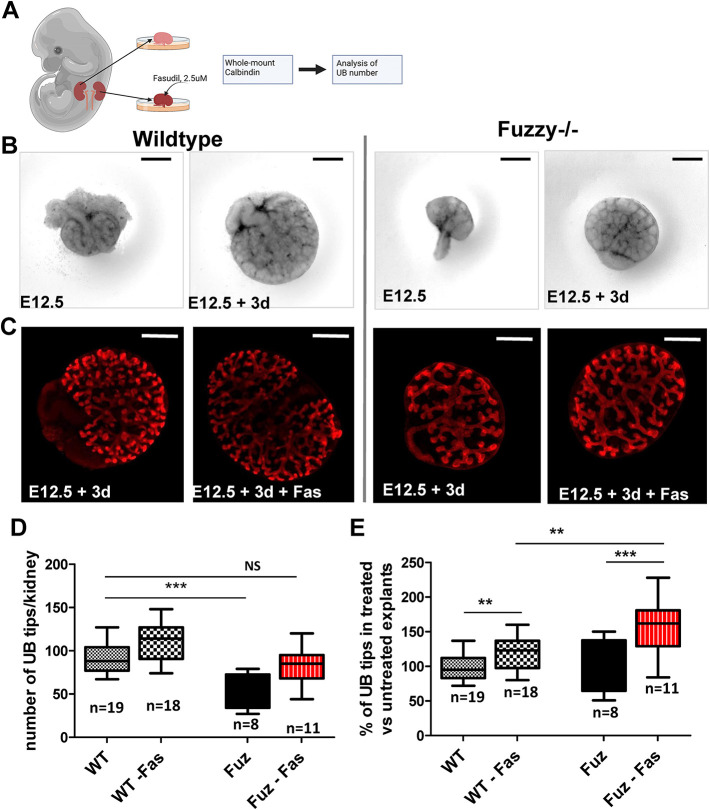
**Effect of fasudil on kidney morphogenesis *ex vivo*.** (A) Schematic of kidney explant experiments; note that one kidney from the same embryo is treated and the other is used as internal control (created using BioRender.com). (B) Phase contrast images of untreated control and *Fuzzy^−/−^* kidneys at the start of the experiment at E12.5 and after 3 days (3d) incubation *ex vivo*. (C) Representative confocal images of control and *Fuzzy^−/−^* treated and untreated kidney explants where branching UB was visualized by whole-mount immunostaining with anti-calbindin antibody (red). (D) UB tip number measurements in control and *Fuzzy^−/−^* explants after a 3-day incubation with or without 2.5 µM fasudil. (E) Percentage of change in UB tip numbers in treated and untreated control and mutant explants. *n* is the number of explants analyzed for each condition. **P≤0.01, ***P≤0.0001 (unpaired two-tailed *t*-test). NS, not significant. The upper and lower limits of the boxes in D and E represent the first quartile (lower end) and the third quartile (upper end); whiskers represent minimum to maximum data points, and the horizontal line represents the median. Scale bars: 150 µm (B); 200 µm. (C).

We recently reported that renal hypoplasia in *Fuzzy^−/−^* mice is likely caused, at least in part, by dysregulation of several cilium-dependent signaling pathways (Wang et al., 2021). Because fasudil rescued ciliogenesis in mutant *Fuzzy* cells and tissues *in vitro* and *ex vivo* (Figs 2 and 3), we analyzed kidney size and UB branching in wild-type and *Fuzzy^−/−^* kidney explants treated with fasudil (Fig. 9A-C). UB tip number in the untreated *Fuzzy^−/−^* kidney explants after 3 days in culture was appreciably lower in comparison with wild-type explants (average of 93 UB branch tips in wild-type versus 53 UB tips in *Fuzzy^−/−^* explants) (Fig. 9D,E). Treatment with 2.5 µM fasudil led to a mild increase in the number of UBs from 93 to 110 (119.2%) in wild-type explants. In contrast, fasudil treatment increased UB tip number in the *Fuzzy^−/−^* explants by 161.2% from 53 to 85 (Fig. 9C-E), indicating a remarkable capacity of the ROCK inhibitor to almost fully rescue UB branching in *Fuzzy^−/−^* kidneys with only a minor effect on wild-type tissue.

## DISCUSSION

**Fig. 10.**
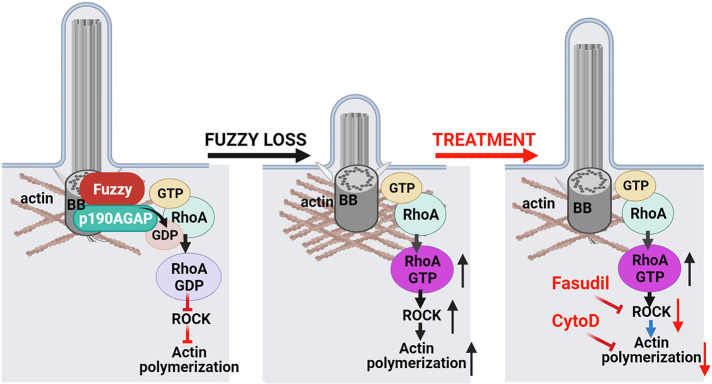
**Model of Fuzzy action during formation of the primary cilium.** We propose that the CPLANE protein Fuzzy binds to and recruits to the basal body the major mammalian actin regulator p190A. At the basal body, p190A normally restricts RhoA activity thereby ‘keeping in check’ downstream activation of ROCK and actin polymerization. This favors primary cilium elongation. In the absence of Fuzzy, p190A is mostly lost at the base of the cilium, causing excessive RhoA activation and derangement of the actin network (depicted by long thin black arrows); this, in turn, interferes with ciliogenesis. Ciliary dysfunction affects cilium-controlled signaling pathways resulting in congenital malformations of ciliopathy spectrum, e.g. skeletal abnormalities, neural tube defects or hypo-dysplastic kidneys. Treatment with inhibitors of Rho kinase (e.g. fasudil), and of actin polymerization (e.g. cytochalasin D) normalizes the actin network at the base of the cilium (depicted by long red arrows) and rescues ciliogenesis in the absence of both Fuzzy and p190A, despite elevated RhoA activity at the ciliary base and ROCK activation (long black arrow). Created in Biorender.com.

Primary cilia are crucial for embryonic development and adult tissue homeostasis (Hildebrandt et al., 2011). Here, we demonstrate that the CPLANE protein Fuzzy is a crucial mediator of ciliogenesis and its loss induces ciliopathy-related phenotypes, including neural tube defects and renal cystic anomalies in mouse embryos. We establish that Fuzzy controls ciliogenesis, at least in part, by regulating actin polymerization at the ciliary base via p190A (Fig. 10). We also show that ciliary and kidney defects in *Fuzzy* mutant cells and tissues can be rescued by ROCK inhibitors.

### Fuzzy and actin regulation at the base of primary cilium

Mutations in CPLANE genes affect primary cilia and ciliary signaling and cause severe ciliopathies in mice (Gray et al., 2009; Heydeck et al., 2009; Seo et al., 2011; Cui et al., 2013; Zeng et al., 2010) and humans (Zhang et al., 2018; Toriyama et al., 2016; Barrell et al., 2022). CPLANE proteins control ciliogenesis via several mechanisms but mainly by organizing cargo protein vesicle traffic to the basal body (Toriyama et al., 2016; Zilber et al., 2013; Adler and Wallingford, 2017; Langousis et al., 2022). Interestingly, in *Drosophila*, Fuzzy and other PCP effectors act as negative regulators of actin polymerization, precluding excessive generation and random positioning of actin-based trichomes on wing cells (Gubb and Garcia-Bellido, 1982). Recent evidence suggests that actin polymerization and branching negatively affect the formation of primary cilium by interfering with cilium-targeted vesicle traffic and plasma membrane remodeling (Copeland, 2020; Kim et al., 2010; Hoffman and Prekeris, 2022; Smith et al., 2020). Furthermore, cortical actin clearing is an obligatory step prior to basal body docking and primary cilium formation (Jewett et al., 2021); failure of cortical actin clearing hinders ciliogenesis (Jewett et al., 2021). Thus, we wondered whether Fuzzy mediates the actin remodeling needed for primary cilium assembly in mammalian cells. Our findings indicate that RhoA activity at the basal body of unciliated *Fuzzy^−/−^* MEFs is excessive and that ROCK inhibitors rescue ciliogenesis in both *Fuzzy* mutant fibroblasts and explant kidneys, suggesting that Fuzzy is a crucial negative regulator of actin polymerization during formation of mammalian primary cilia. Similarly, in a study by Hernandez- Hernandez et al., loss of *Bbs4* was reported to increase F-actin and RhoA activity, and to reduce the number of ciliated cells; the latter was rescued by treatment with ROCK inhibitors (Hernandez-Hernandez et al., 2013). Together, published studies and our data confirm that ciliopathy-causing proteins may act as negative actin regulators at the base of the primary cilium, although the mechanistic aspects remain largely unknown.

Interestingly, Park et al. previously demonstrated that *Xenopus* multiciliated ectodermal *fuz* morphant cells are marked by a decrease in cortical actin accumulation (Park et al., 2006); this observation appears to contrast with our findings. The discrepancy in actin behavior between the multiciliated *Xenopus* cells and our findings might be attributed to potential differences in actin requirements for assembly and functions of primary versus motile cilia. In multiciliated cells, docking and planar polarization of numerous basal bodies are highly dependent on the presence of a dense cortical actin network (Park et al., 2008; Herawati et al., 2016). Thinning of cortical actin is associated with abnormal docking and randomized polarization of basal bodies, precluding normal ciliogenesis and directional ciliary beating (Werner et al., 2011). During formation of primary cilium, the crosstalk between actomyosin contraction (mediated by RhoA pathway) and microtubule stabilization generates the force that drives the basal body toward the apical membrane – for this, substantial actomyosin activity is required (Copeland, 2020). Unlike multiciliated cells, the subsequent basal body docking, formation of ciliary vesicle and initial steps of primary cilium assembly require cortical actin clearing and plasma membrane remodeling (Jewett et al., 2021). We detected increased F-actin density around basal bodies in tubular epithelial cells in embryonic kidneys, which could reflect failure of cortical actin clearing due to *Fuzzy* mutations in renal cells *in vivo*. Our observations that p190A is mislocalized from the basal body in *Fuzzy^−/−^* MEFs, that its overexpression cannot rescue ciliogenesis in the absence of Fuzzy, and that there is biochemical interaction between Fuzzy and p190A, together suggest the importance of Fuzzy to recruitment of p190A at the ciliary base. Given that p190A powerfully suppresses RhoA GTPase activity (Stewart et al., 2016), the increased RhoA activity at the basal body in *Fuzzy^−/−^* MEFs could be attributed to the loss of p190A-mediated RhoA inactivation. Notably, inactivating *ArhGAP35* mutations in mice also lead to shortened primary cilia, excessive actin accumulation and inappropriate RhoA activation at the basal body in renal epithelial tubule cells (Stewart et al., 2016).

Treatment of kidney explants with fasudil *ex vivo* rescued both ciliogenesis and UB branching. This implies that defective ciliary function may cause congenital nephron deficit and renal hypoplasia. Indeed, primary cilia have been implicated in several signaling pathways regulating UB branching (May-Simera and Kelley, 2012; Gill and Rosenblum, 2006; Kunova Bosakova et al., 2019). Our recent RNA-sequencing and *in situ* hybridization studies of *Fuzzy^−/−^* kidneys uncovered deregulation of multiple cilia-dependent pathways, including Shh (Wang et al., 2021). Thus, fasudil may correct UB branching in *Fuzzy* mutant cells by restoring normal cilial signaling.

Interestingly, the effect of fasudil on UB branching appears to be more powerful than its effect on ciliary length: UB number in fasudil-treated explants was fully normalized, whereas ciliary length was still significantly shorter than in wild-type tissue. Tubulogenesis in many organs, including lungs and kidney, relies on collective cell movements controlled by PCP (Yates et al., 2010; Kunimoto et al., 2017; Derish et al., 2020). Consistent with this idea, we previously reported that defective collective cell movements in *Fuzzy^−/−^* MEFs accompanied the loss of planar polarization (Zilber et al., 2013). Thus, it is plausible that Fuzzy regulates UB branching via cilia-dependent and -independent mechanisms. Interestingly, regardless of the mechanisms involved, ROCK inhibitors rescue both ciliogenesis and kidney organogenesis in *Fuzzy* mutants.

### Genetic interactions between *Fuzzy* and *ArhGAP35*

Our observations show clear genetic interactions between *Fuzzy* and *ArhGAP35* genes. A characteristic of human ciliopathies is the presence of anomalies that involve multiple organs (Hildebrandt et al., 2011). Our data suggest that the severity of malformations in multiple organs is also an oligogenic trait related to loss of *Fuzzy* and *ArhGAP35* alleles. In our colony, we never detected any phenotypes in *Fuzzy* or *ArhGAP35* heterozygotes; however, ∼3% of *Fuzzy^+/−^;ArhGAP35^+/D34^* compound heterozygotes (loss of two of the four alleles) exhibited exencephaly. *Fuzzy^+/−^;ArhGAP35^D34/D34^* mice (loss of three of the four alleles) displayed spina bifida and microphthalmia. Although spina bifida has been reported in a small percentage of *ArhGAP35^D34/D34^* mice (Stewart et al., 2016), we never saw microphthalmia in either *Fuzzy^+/−^* or *ArhGAP35^D34/D34^* animals. Interestingly, mice with a full knockout of the *ArhGAP35* gene display variably penetrant microphthalmia (Brouns et al., 2000), and *ARHGAP35* variants have recently been identified in humans with eye defects (Reis et al., 2022). Thus, our results indicate that loss of one *Fuzzy* allele strongly affects penetrance of eye phenotype in *ArhGAP35^D34/D34^* mice. Double-homozygous mice (loss of all four alleles) exhibited 100% of exencephaly, microphthalmia, polydactyly, and severe developmental delay, consistent with defects observed in the ciliopathy spectrum (Hildebrandt et al., 2011). In agreement with this, we also discovered a combined effect of loss of *Fuzzy* and *ArhGAP35* alleles on ciliary length and the percentage of ciliated cells: loss of three of the four alleles affected ciliogenesis with more severity than cilia in single homozygotes. Complete loss of both *Fuzzy* and *ArhGAP35* alleles resulted in few short cilia. Thus, the ciliogenesis and development of multiple organs may depend on cooperative effects of *Fuzzy* and *ArhGAP35* in regulating basal body actin accumulation, and the effect on cilia formation and the penetrance of organ-specific phenotypes may be a function of *Fuzzy* and *ArhGAP35* gene dosage.

The strong genetic interactions between *Fuzzy* and *ArhGAP35* is also seen in optimization of UB branching and kidney size during embryogenesis. Importantly, renal hypoplasia due to *Fuzzy* and *ArhGAP35* interactions appears to have a strong oligogenic trait. Thus, moderately severe renal hypoplasia in *Fuzzy^−/−^* homozygotes was worsened in *Fuzzy^−/−^* embryos lacking one *ArhGAP35* allele (loss of three of the four alleles). Similarly, renal hypoplasia among *ArhGAP35^D34/D34^* embryos was worsened by the loss of one *Fuzzy* allele (loss of three of the four alleles) to the point that 4/6 examined embryos were anephric. Similarly, very severe renal hypoplasia was seen in double homozygotes (loss of all four alleles). Two out of three *Fuzzy^−/−^;ArhGAP35^D34/D34^* mice had only residual kidney rudiments and the third lacked kidneys, suggesting synergistic effects on kidney development when three out of four alleles or both genes were lost. Thus, Fuzzy and p190A proteins appear to cooperate in a final common pathway that optimizes primary cilial function during branching morphogenesis of the kidney. The severity of the renal phenotype depends on the gene dosage of the two aforementioned genes.

In summary, this study establishes a previously unappreciated role for CPLANE protein Fuzzy in controlling actin assembly at the primary cilium via interactions with p190A. It suggests a potential new treatment with ROCK inhibitors, which rescue both the ciliary defects and renal hypoplasia. Therapeutic treatments for ciliopathies are limited and have been focused on improving symptoms rather than treating the causes. Our study offers a proof-of-principle therapeutic strategy for the severe human developmental defects caused by CPLANE gene mutations.

## MATERIALS AND METHODS

### Ethics statement

All experiments were approved by the McGill Animal Care Committee (protocol MUHC-7606) and conducted according to Canadian Council of Animal Care ethical guidelines.

### Mouse breeding and husbandry

The generation of *Fuzzy* transgenic mice from the gene-trap embryonic stem cell line AN0439 was previously described (Seo et al., 2011). Insertion of the βGeo gene-trap cassette in the third intron of the *Fuzzy* gene disrupts the wild-type allele creating a loss-of-function (null) mutation. *Fuzzy^−/−^* embryos were generated by brother-sister mating of heterozygous *Fuzzy^+/−^* mice. The plug on the morning after mating was considered E0.5. Pregnant females were sacrificed at E12.5-E14.5 because the majority of *Fuzzy^−/−^* mice in our colony do not survive past E14.5. E16.5 embryos were produced in Dr Karen Liu's laboratory (King's College London, UK), using *Fuz^Gt1(neo)^* mice generated previously on a mixed background (Gray et al., 2009).

The heterozygous *ArhGAP35^+/D34^* mice were initially described by Stewart et al. (2016). The mutant mouse bears a Leu1396 to Gln substitution in the GAP domain of the *ArhGap35* gene, rendering the p190A protein non-functional. Mutant *ArhGAP35^+/D34^* mice were on a C57/Bl6 background.

*Fuzzy^+/−^* mice were crossed with *ArhGAP35^+/D34^* mice to generate double-heterozygous *Fuzzy^+/−^;ArhGAP35^+/D34^* mice. The heterozygotes were viable, fertile, and lacked any discernable phenotype. Embryos with various gene dosages for *Fuzzy* and *ArhGAP35* genes were generated by crossing *Fuzzy^+/−^;ArhGAP35^+/D34^* male and female mice. Pregnant mice were sacrificed at E14.5, and embryos were collected.

### Genotyping

All genotyping procedures were performed with DNA samples isolated from mouse tail samples (adults) or limb samples (embryos) using a M-Fast PCR Genotyping Kit (ZmTech Scientifique, GT-003/GT-003p) as described by the manufacturer. Genotyping was performed with the following primers: *Fuzzy* wild-type allele (500 bp), mFuzzy-Exon3-Forward primer 5′-CACCTCTGAGCTGAGGCTGG-3′, mFuzzy-Exon4-Reverse primer 5′-CTCAATTCTTTCTTCAGTCTTTC-3′; *Fuzzy* gene-trapped allele (680 bp): bGEO-1F primer 5′-TTATCGATGAGCGTGGTGGTTATGC-3′, bGEO-2R primer 5′-GCGCGTACATCGGGCAAATAATATC-3′. *ArhGAP35^+/D34^* primers encompassing the mutation were used (874 bp): Primer1-F 5′-TGATTGTGGATGTGGTAGGCG-3′, Primer2-R 3′-GCTTCTCACGGCAACATGGAAC-5′. *Fuz^Gt1(neo)^* mice were genotyped as previously described (Tabler et al., 2013).

The PCR mixture included 1×PCR buffer, 1.5 mM MgCl_2_, 250 μM dNTP mix, 5 μl 10 mM forward primer, 0.5 μl 10 mM reverse primer, 0.2 μl Taq polymerase, and water to a total volume of 20 μl. All the PCR reagents were from Life Technologies. PCR conditions were: Step 1, heat activation at 94°C for 5 min; Step 2, denaturation at 94°C for 30 s; Step 3, annealing at 60°C for 45 s; Step 4, extension at 72°C for 1 min. Steps 2-4 were repeated for 34 cycles followed by the final elongation at 72°C for 5 min. The PCR fragments were detected on 1% agarose gel by staining with 6x Fluo-DNA Loading Buffer (ZmTech Scientifique) alongside Fluo-DNA ladder (ZmTech Scientifique). *ArhGAP35* PCR fragments were visualized on the 1% agarose gel to confirm that only a single fragment was present and then genotyped by the Génome Québec service centre.

### Tissue culture

Generation of wild-type, *Fuzzy^−/−^* and *ArhGap35^D34/D34^* MEFs was previously described (Seo et al., 2011; Stewart et al., 2016). MEFs were plated on 15 mm glass coverslips (Thermo Fisher Scientific) coated with rat Collagen I (Life Technologies) at 10^5^ cells per well in 12-well plates (Sarstedt). DMEM/F12 growth medium was supplemented with 10% fetal bovine serum (FBS), 1% non-essential amino acids (NEAA) and 1% penicillin/streptomycin (all from Wisent). After 24 h, cells were starved in serum-free medium for another 24 h to induce ciliogenesis and then treated with actin polymerization inhibitors for 8 h. Fasudil was diluted to 50 µM in PBS, Y27632 diluted to 1 µM, and cytochalasin D to 0.5 µM in DMSO (all inhibitors from Sigma-Aldrich). Inhibitor concentrations were optimized to ensure high cell viability using Cell Counting Kit - 8 reagent (Sigma-Aldrich) according to manufacturer recommendations. At the end of the incubation period, cells were washed with DMEM and fixed with 4% paraformaldehyde in PBS (PFA/PBS) for 15 min after four 3-min washes with PBS. For colocalization experiments, both *Fuzzy^−/−^* and *ArhGap35^D34/D34^* MEFs were grown under similar conditions. Human embryonic kidney HEK293T cells were grown in 100 mm Petri dishes in DMEM supplemented with 10% FBS and 1% streptomycin/penicillin until ready for transfection.

### Tissue collection and processing

Collected embryos were washed in cold PBS and visually inspected for macro-defects (size, overall development, neural tube defects, polydactyly, eye abnormalities) and photographed. Embryos were then processed either as frozen tissues or for paraffin embedding. To freeze the tissues, E14.5 embryos were fixed in 4% PFA/PBS for 2 h and then incubated at 4°C with 15% sucrose in PBS for 2 h followed by 30% sucrose in PBS until the tissues descended to the bottom of the vials. Embryos were positioned sagittally in the molds and frozen in O.T.C. Compound (Tissue-Tek from Fisher Scientific) in a methylbutanol/dry-ice bath. Cryoblocks were stored at −80°C until use. The cryoblocks were sectioned at 5 or 10 µm on a cryostat (Leica Biosytems).

For paraffin-embedding, the embryos were fixed overnight at 4°C in 4% PFA/PBS, washed in PBS, dehydrated in a mixture of ethanol/PBS of various concentrations, and embedded in paraffin blocks by the McGill University Health Centre Research Institute Histology service. The blocks were stored at room temperature (RT) until use. The paraffin blocks were sectioned at 4 μm in the sagittal plane on a rotary microtome (Leica Biosystems). The kidneys were identified in paraffin sections using a ×5 magnification light microscope and transferred to microscope slides (Thermo Fisher Scientific). The sections were dried at 37°C overnight and stored at RT until use.

### Explant culture *ex vivo*

E12.5 embryos from *Fuzzy^+/−^* timed pregnancies were micro-dissected to harvest both kidneys with a Zeiss Discovery V.8 stereo microscope (Zeiss). The kidneys were cleaned of surrounding tissues and positioned atop the 0.4 µm 30 mm diameter insert (Sigma-Aldrich) in 35 mm Petri dishes containing DMEM/F12, 25 mM HEPES supplemented with 2% FBS, 1% NEAA, 2 mM GlutaPlus, 1% penicillin/streptomycin, 0.615 μg/ml amphotericin (all from Wisent), 10 ng/ml T3, 36 ng/ml hydrocortisone and 50 ng/ml PGE1 (all from Sigma-Aldrich), 5 ng/ml hEGF and 1× ITS (both Thermo Fisher Scientific). The kidney explants were incubated in growth medium in the presence or absence of 2.5 µM fasudil (Sigma-Aldrich) for 72 h. The two kidneys from each embryo were separated into ‘treated’ and ‘untreated’ groups. The medium±fasudil was changed every 24 h. A minimum of 8-11 explants were used per genotype for each treatment condition.

### Immunofluorescence

To visualize cilia in MEFs, the cells were fixed in 4% PFA/PBS for 15 min at RT, washed three times with PBS and permeabilized for 5 min with 0.5% Triton X-100 in PBS solution. Blocking was carried out in 3% bovine serum albumin (BSA), 3% normal goat serum (NGS), 3% normal donkey serum (NDS) (both sera from Life Technologies) in PBS for 30 min, then blocked with donkey anti-mouse IgG Fab (1:500; Jackson ImmunoResearch, 415-007-003) for 30 min and incubated with anti-acetylated-α-tubulin mouse antibody (1:2000; Sigma-Aldrich, T6793) in 1% BSA in PBS for 1 h 30 min. Acetylated-α-tubulin was visualized with donkey Alexa Fluor 488 anti-mouse antibody (1:1000; Thermo Fisher Scientific, A-21202) in 1% BSA in PBS for 1 h. All procedures were performed at RT. The cells were washed and incubated for 2 min with 4′,6-diamidino-2-phenylindole dihydrochloride (DAPI; Invitrogen; 1:500) in 0.1% Tween 20 in PBS at RT and mounted with Fluoromont-G (Life Technologies).

The paraffin-embedded E14.5 and E16.5 sections were de-paraffinized and hydrated using standard protocols. Antigen retrieval was performed in boiled Antigen Unmasking solution (H-3300, Vector Laboratories) for 20 mins in a microwave at power setting 2 (Toshiba). The slides were cooled down for 30 min at RT, washed in PBS for 10 min and permeabilized in 1% Triton X-100 in PBS for 1 h at RT. The sections were blocked with 3% BSA (BioShop Canada), 5% NGS, 5% NDS (Jackson ImmunoResearch), 0.2% Triton X-100 in PBS for 1 h at RT. The slides were then incubated at 4°C overnight with anti-calbindin D-28K antibody (1:300; Calbiochem, PC253L) or anti-ARL13B rabbit antibody (1:100; Proteintech, 17711-1-AP) in in 3% BSA, 3% NGS, 3% NDA, 0.01% Triton X-100 in PBS at 4°C overnight. The slides were washed four times in PBS for 5 min each and incubated with Alexa Fluor 568 donkey anti-rabbit mouse IgG (H+L) secondary antibody (Jackson ImmunoResearch) for 1 h at RT in the dark. Slides were washed four times in PBS, incubated for 7 min in DAPI, and mounted with ProLong™ Gold Antifade mounting medium (Invitrogen Molecular Probes). In some experiments, fluorescently tagged Lotus Tetragonolobus Lectin (LTL) LTA (a marker of proximal tubules; 1:200; Vector Laboratories, FL-1321-2 was used. All fluorescence images were acquired on a Zeiss Axio Observer 100 microscope with a Zeiss AxioCam MRm monochrome camera (calbindin) or *z*-stacks of cilia images were acquired using a Zeiss LSM880 confocal microscope.

### Wholemount immunostaining

To visualize UBs, the explants were fixed in cold methanol for 30 min, washed twice with cold PBS, permeabilized in 0.1% Tween 20 in PBS for 30 min, and blocked in 5% BSA, 0.2% skim milk (BioShop Canada), 0.5% Triton X-100 for 1 h on ice. The explants were washed with 0.1% Tween 20 in PBS for 30 min and incubated with anti-calbindin antibody (1:100) in 5% BSA, 0.2% skim milk, 0.3% Triton X-100 for 20 h at 4°C, followed by four washes in 0.1% Tween 20 in PBS and incubation with donkey Alexa Fluor 568 anti-rabbit antibody (1:100; ThermoFisher, A10042) and DAPI (1:100) in 5% BSA, 0.2% skim milk, 0.3% Triton X-100, 5% NDS for 20 h. Finally, the explants were washed four times with 0.1% Tween 20 in PBS, three times in PBS and mounted with Fluoromount-G (ThermoFisher, 00-4958-02).

To visualize cilia, explants were washed in PBS and fixed in 4% PFA in PBS on ice for 2 h. The tissues were then additionally fixed in cold acetone for 10 min, quickly washed with PBS and twice with 0.1% Triton X-100 in PBS and incubated for 30 min in 0.5% Triton X-100 in PBS. Explants were pretreated for 1 h with the AffiniPure Fab Fragment donkey anti-mouse (1:500; Jackson ImmunoResearch, 415-007-003) in 5% BSA, 0.2% skim milk, 0.5% Triton X-100 in PBS and then washed in cold PBS containing 0.1%, 0.5% and lastly 0.3% Triton X-100 for 15 min each wash. Explants were blocked in 5% BSA, 0.2% skim milk, 0.3% Triton X-100 and then incubated with anti-ARL13B (1:100) in blocking buffer for 20 h at 4°C. The tissues were washed four or five times with 0.1% Tween 20 in PBS and then incubated with goat Alexa Fluor 488 anti-rabbit antibody (1:150) and DAPI in 5% BSA, 0.2% skim milk, 5% NGS, 0.3% Triton X-100 for 20 h at 4°C in darkness. The final washes and mounting were carried out in ice-cold PBS containing 0.1%, 0.5% and 0.3% Triton X-100 for 15 min per wash. and then mounted with Fluoromount-G (ThermoFisher, 00-4958-02). *Z*-stacks of whole tissue were acquired using a Zeiss LSM880 confocal microscope.

### Measurements of ciliary length

Methodical imaging of the entire coverslip was employed to generate a minimum of ten images for each coverslip for each experimental condition. Three independent experiments were performed. *Z*-stack confocal 0.25 μm slices were obtained for each field of cells or tissues to cover the length of 99% of visible cilia. The 0.25 μm distance was established empirically in the preliminary experiments to increase accuracy of measurements. Deconvoluted images were used to measure ciliary length manually using ImageJ software (Fiji) in the cells or tissues immunostained with ARL13b or anti-acetylated-α-tubulin antibody and co-stained with DAPI to label nuclei.

### Colocalization assays

Wild-type, *Fuzzy^−/−^* or *ArhGap35^D34/D34^* MEFs were grown on collagen-coated coverslips in 6-well plates for 24 h. The cells were transfected with a 1:1.5 ratio of DNA:Lipofectamine 3000 as recommended by the manufacturer (Life Technologies) using 2.5 μg GFP-p190A (Stewart et al., 2016) or GFP-hFuzzy (Zilber et al., 2013). The cells were incubated for 24 h post-transfection in DMEM supplemented with 20% FBS at 37°C, washed with DMEM and starved for 24 h in growth medium with 0.5% serum to induce ciliogenesis. The cells were fixed for 15 min with 4% PFA in PBS, washed with PBS, and permeabilized with 0.5% Triton X-100 in PBS for 30 min. The cells were preincubated with Fab donkey anti-mouse (1:500; Jackson ImmunoResearch, 415-007-003) in PBS for 30 min, washed in 0.1% Triton X-100 in PBS three times and incubated with anti-γ-tubulin antibody (1:4000; Sigma-Aldrich, T6557) and anti-Arl13B antibody (1:100) in 5% NDS, 0.1% Triton X-100 in PBS for 2 h at RT, followed by incubation with Cy3 anti-mouse antibody (1:800; Jackson ImmunoResearch, 715-167-003) and goat Alexa Fluor 647 anti-rabbit antibody (1:250; Jackson ImmunoResearch, 111-605-003) in 5% BSA, 0.2% skim milk, 5% NGS, 0.3% Triton X-100 for 1 h at RT. The coverslips were mounted in Fluoromount-G. Images were acquired using a Zeiss LSM880 confocal microscope. The same pinhole and channel intensity were used for Cy3 and GFP channels. Zen Black software was used to quantify the localization coefficient. Three experiments per cell type were performed.

The Rho activity reporter pGFP-rGBD (Addgene #26732; Benink and Bement, 2005) was transfected into wild-type and *Fuzzy^−/−^* MEFs with Lipofectamin 3000 (ThermoFisher, L3000150). The cells were fixed 24 h post-transfection as above. Both basal bodies and cilia were detected by simultaneous incubation with anti-γ-tubulin antibody (1:4000) and anti-ARL13B antibody (1:250), respectively, visualized with secondary donkey Alexa 647 anti-mouse antibody (1:1000; Jackson ImuunoResearch, 715-605-151) and goat Alexa Fluor 568 anti-rabbit (1:1000; ThermoFisher, A10042), respectively. The cells were washed and mounted as above. In some experiments, Cherry or Cherry-Fuzzy expression vectors (published by Zilber et al., 2013) were co-transfected with pGFR-rGBD. The cilia were visualized using anti-acethylated-α-tubulin antibody (1:1000; Sigma-Aldrich, T6793), the basal body was detected using anti-γ-tubulin antibody (1:4000; Sigma-Aldrich, T6557), and both primary antibodies were visualized using Alexa Fluor 647 AffiniPure donkey anti-mouse IgG (H+L) antibody (Jackson ImmunoResearch, 715-605-151) in order to see both structures in the same channel. The images were acquired on a Zeiss LSM 880 confocal laser scanning microscope. The intensity of pGFP-rGBD expression at the basal body was measured using Zen Black software in a designated 1.45 µm^2^ area centered on the basal body (visualized with anti-γ-tubulin antibody). To account for the differences in the GFP-rGBD transfection in analyzed cells, the intensity of the GFP-rGBD was also measured in the cytoplasm adjacent to the basal body; three separate 1.45 µm^2^ areas were designated within the circular area around the basal body and measurements of these areas were obtained (as depicted in Fig. S5A). The pGFP-rGBD intensity at the basal body was normalized to the average intensity of three cytoplasmic areas in pGFP-rGBD-transfected cells. Three independent experiments were carried out.

### Actin analysis in renal tubules

E14.5 frozen wild-type and *Fuzzy^−/−^* embryos were sectioned at 10 µm, and renal proximal tubules were identified by LTA staining. Basal bodies were visualized with anti-γ-tubulin anti-mouse antibody (Sigma-Aldrich). F-actin filaments were stained with Alexa Fluor 565-conjugated phalloidin (Sigma-Aldrich, 94072). Imaging was performed on a Zeiss LSM880 laser scanning confocal microscope and images were analyzed with Zen 11 software.

### Morphological analysis of embryonic kidneys

The LTA-stained images of the E16.5 kidney sections of wild-type and *Fuzzy^−/−^* embryos were analyzed by measuring the length and thickness of each tubule cross-section. Only the tubule cross-sections with a length/width ratio between 0.8 and 1.2 were used for analysis. The cross-sectional area was measured, and the cell nuclei in each structure were counted. The glomeruli were identified by morphology; the areas of Bowman's capsule and the tuft in each glomerulus were measured using the contour application in Zen lite software (Zeiss, Germany).

To assess kidney morphology and the number of UBs in the E14.5 embryos with various *Fuzzy/ArhGAP35* allele dosages, the ‘maximal’ sagittal kidney sections (in which collecting ducts could be traced from the center to the cortical layer) were identified and analyzed. The number of UB tips per maximal section visualized with anti-calbindin antibody (1:100) was counted; two maximal sections per kidney were analyzed. For most genotypes six to ten embryos were processed and the kidney sections were analyzed. For the wild-type and double-homozygous *Fuzzy^−/−^;ArhGAP35^D34/D34^* genotype, three embryos were analyzed per genotype. The wild-type and *Fuzzy^−/−^* kidney explants were stained with calbindin antibody as wholemount tissues, and all UB tips in each tissue were counted. Eight to 19 explants per condition per genotype were analyzed.

### Hematoxylin and Eosin staining

Paraffin-embedded embryonic sections were deparaffinized and hydrated in decreasing concentrations of ethanol in PBS. The slides were stained in Mayer's Hematoxylin Solution (MHS16, Sigma-Aldrich) for 1-4 min at RT, rinsed in warm running tap water and then in 95% ethanol. The slides were then placed in EosinY alcoholic solution (1:200, HT110116, Sigma-Aldrich):glacial acetic acid for 2 s and rinsed. The slides were immersed in 95% ethanol and xylene substitute (Thermo Fisher Scientific) twice for 3 min each and mounted with mounting medium (Life Technologies). Hematoxylin and Eosin images were acquired using a Zeiss AXIO Imager D2 with Zeiss Axio CAMera ICc5. Zen lite software (Zeiss) was used.

### Co-immunoprecipitation

Confluent HEK293T cells were transfected with 2 µg Fuzzy-Flag cDNA (GenScript) or 2 µg pcDNA3.1 (Life Technologies) and 1.5 µg p190A-GFP cDNA (Stewart et al., 2016) using Lipofectamine 3000 (Invitrogen) as recommended by the manufacturer. The cells were washed in cold PBS and lysed 24 h post-transfection in 500 µl lysis buffer: 50 mM HEPES, pH 7.5, 150 mM NaCl, 10% glycerol, 0.5% Triton X-100, 1.5 mM MgCl_2_, 1 mM EGTA, 25 mM NaF, 2 mM Na_3_VO_4_, 10 mM sodium pyrophosphate (ThermoFisher Scientific) 1× Protease Inhibitor Cocktail (BioShop Canada), 1 mM phenylmethylsulfonyl fluoride; all inhibitors were from Sigma-Aldrich. The cells were scraped in the lysis buffer into vials and lysed on ice for 20 min with vigorous intermittent vortexing. The cell lysates were precleared by centrifugation at 14,000 rpm (18,400 ***g***) at 4°C for 15 min. The protein concentration in each tube was measured by the Bradford method. A minimum of 50 µl of each protein lysate was saved as ‘input’ fraction. Three-hundred microliters of the protein lysates containing 0.75-1 mg of total protein were incubated overnight at 4°C with 1:50 dilution of either anti-p190A RhoGAP (D8Q6C, Cell Signaling Technology, 12164S) or anti-FLAG (Sigma-Aldrich, F3165), then 100 µl protein A/G agarose beads (Santa Cruz Biotechnology, sc-2003) or protein A agarose beads (sc-2001, Santa Cruz Biotechnology) were washed in the lysis buffer overnight and added to the protein/anti-p190 antibody or protein/anti-FLAG antibody mix, respectively, and incubated for 3 h at 4°C with rotation. The protein/antibody/bead complexes were then washed twice in cold washing buffer (50 mM HEPES, pH 7.5, 200 mM NaCl, 10% glycerol, 0.5% Triton X-100, 1.5 mM MgCl_2_, 1 mM EGTA and the same protease inhibitors as in the lysis buffer) and then twice in the washing buffer above with 500 mM NaCl. Beads were collected by centrifugation at 2000 rpm (380 ***g***); 2× sample buffer, containing 5% mercaptoethanol (Thermo Fisher Scientific) was added to each vial. The samples were heated for 10 min at 95-98°C and used for western blotting or stored at −80°C.

### Western immunoblotting

All samples were resolved on 7-8% acrylamide gel using routine techniques. Each co-immunoprecipitation variant and 20 µg of each ‘input’ fraction were resolved in parallel. The proteins from the gels were transferred onto BioTrace NT membrane (VWR). The membranes were blocked in blocking buffer [5% BSA, Tris-buffered saline, pH 8.0, supplemented with 0.1% Tween 20 (TBST)] for 1 h at RT. The co-immunoprecipitation and one of the input membranes were incubated with anti-p190A antibody (1:1000) in blocking buffer overnight at 4°C on a rotating wheel, and the other co-immunoprecipitation and remaining input membranes were incubated with anti-FLAG antibody (1:1000, Sigma-Aldrich, F3165) under the same conditions. All membranes were washed four times for 10 min in TBST buffer and incubated with either HRP goat anti-mouse antibody (1:13,000; ThermoFisher, A16066) to detect anti-FLAG antibody or with HRP-conjugated goat anti-rabbit IgG (H+L) secondary antibody (1:13,000; ThemoFisher, 32460) to detect anti-190A antibody in 5% skim milk in 0.1% Tween 20 (TBST) milk buffer for 1 h at RT. The membranes were then washed in washing buffer and proteins detected using Clarity Western ECL Subs (Bio-Rad).

### Urine analysis

Urine was collected from 8-month-old wild-type and *Fuzzy^+/−^;ArhGAP35^+/D34^* mice and stored at −80°C. Urine albumin levels were determined using the Mouse Albumin ELISA Assay kit (Bethyl Laboratories) following manufacturer recommendations.

### Statistical analysis and data presentation

For quantification of morphological features and cilia, ImageJ software version 4.0 was used (National institute of Health, USA). GraphPad Prism 9.1 software was used to produce graphs. For two group comparisons, a two-tailed, unpaired Student's *t*-test was used. For comparisons among more than two groups, one-way ANOVA was used following by post-hoc analyses using *t*-tests with Bonferroni's correction. All graphs show mean±s.d. *P*<0.05 was considered significant; the *P*-values in the graphs are shown as: *P≤0.05, **P≤0.01, ***P≤0.001 and ****P≤0.0001; non-significant values are not shown.

## Supplementary Material



10.1242/develop.202322_sup1 Supplementary information
