# Supplementary material for: The CPLANE protein Fuzzy regulates ciliogenesis by suppressing actin polymerization at the base of the primary cilium via p190A RhoGAP
Source: Development. 2024 Mar 28;151(6):dev202322. doi: 10.1242/dev.202322 (PMC11006408; doi:10.1242/dev.202322)
Supplement: Supplementary information [file develop-151-202322-s1.pdf]

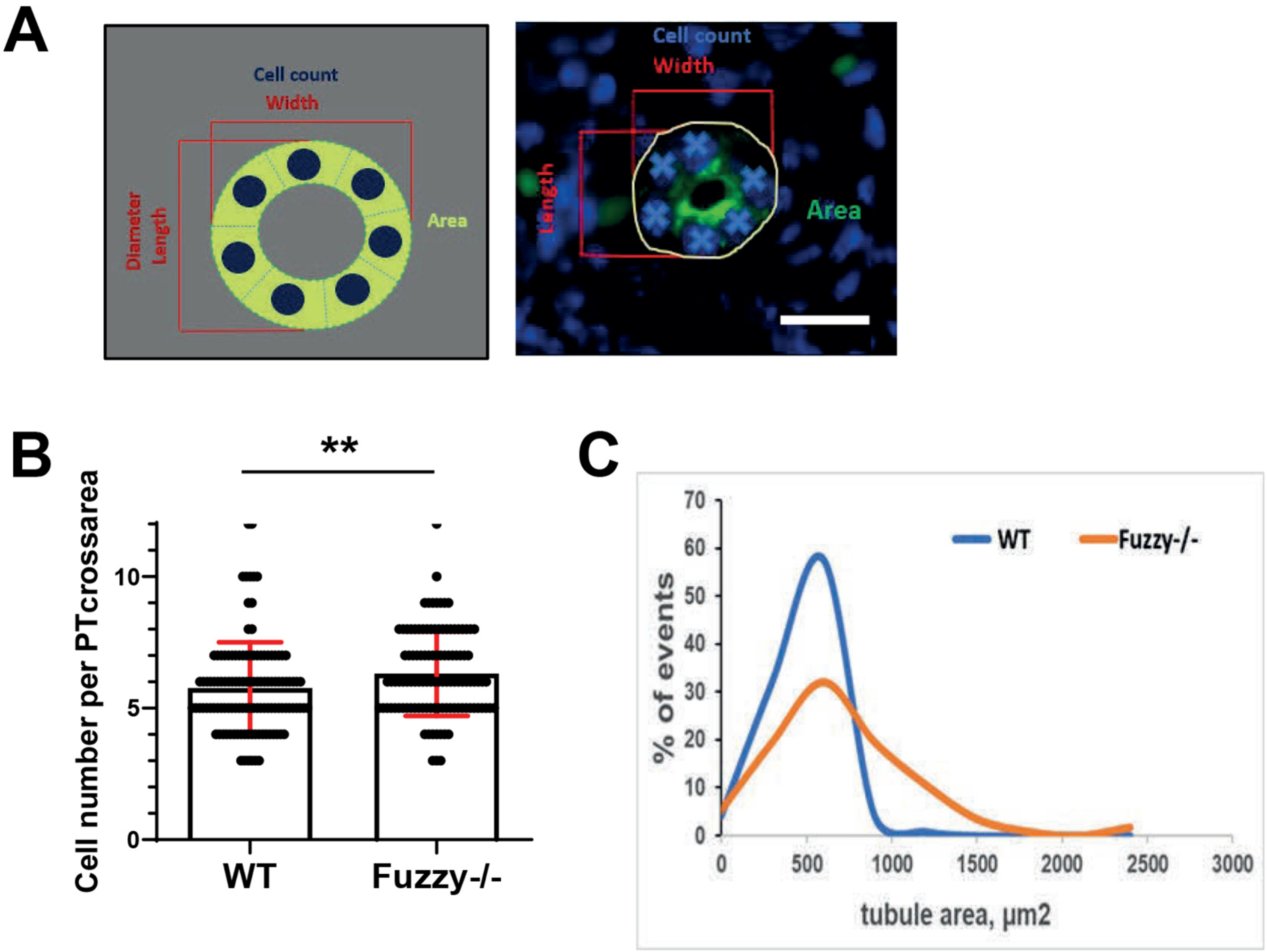

Fig. S1. Analysis of proximal tubule size of E16.5 wildtype and *Fuzzy-/-* kidneys *in vivo.* A. Cartoon (left) and a fluorescence image (right) of E16.5 kidney proximal tubule cross-section. The measurements of width and length are shown, as well as the area and the number of cells (by counting DAPI-stained nuclei). The width and length were used to define the circularity of each cross-section. Only images with W:L between 0.8 & 1.2 were used to measure cross-sectional area (shown in Figure 1); scale bar 10 µm. **B.** Statistical analysis of the number of cells in cross-sectional area: n= 120 (WT) and n= 112 (Fuzzy$^{-/-}$) structures were analyzed in 4 kidneys for each genotype. **C.** Distribution of various area sizes in E16.5 WT and Fuzzy$^{-/-}$ proximal tubules. Each event (bin) is 300 µm2.

Development • Supplementary information

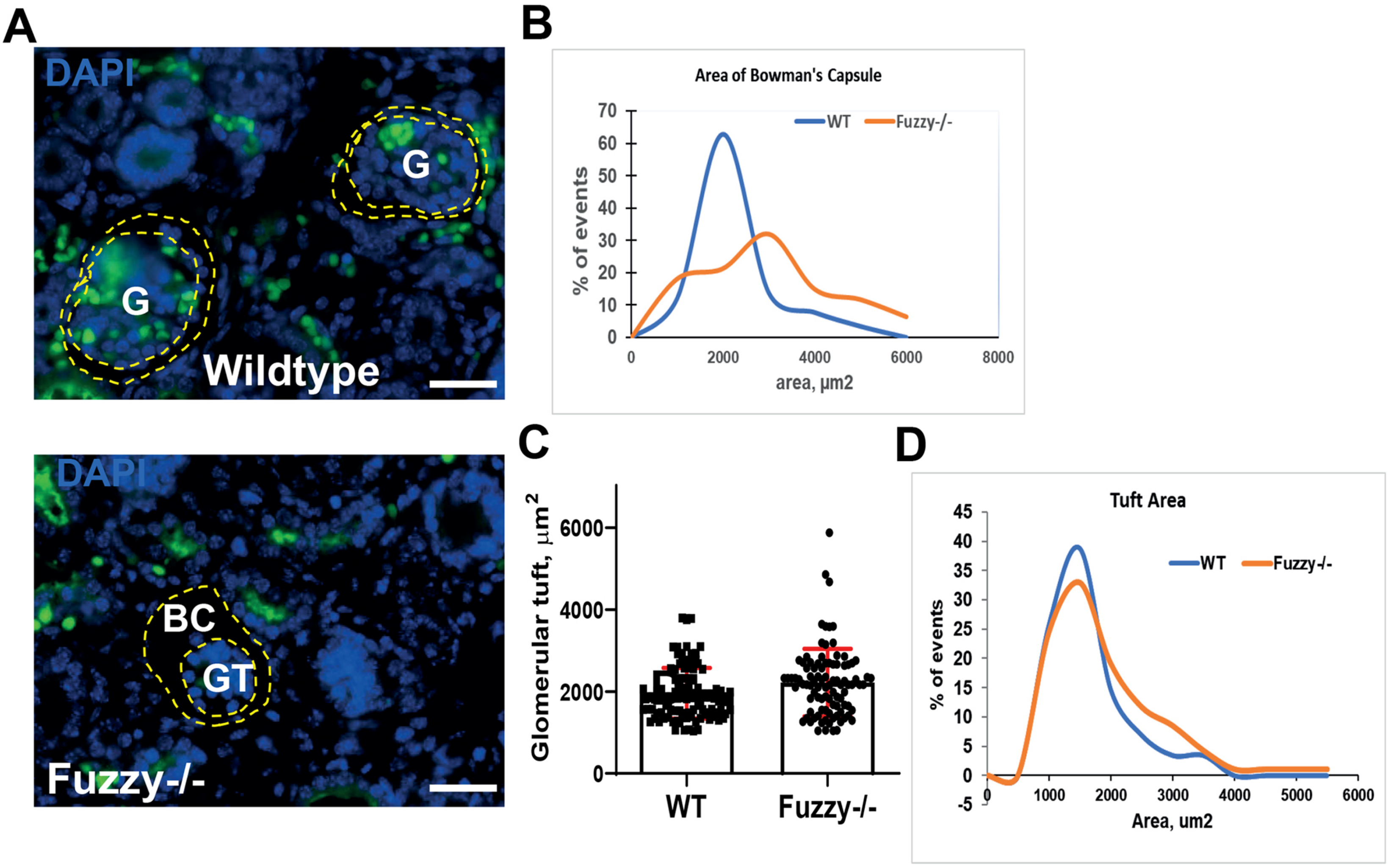

Fig. S2. Glomerular morphology in E16.5 wildtype and *Fuzzy*$^{-/-}$ kidneys. A. Representative images of glomeruli (G) identified by characteristic shape in E16.5 kidneys stained with DAPI. Note, blood cells autofluorescence is seen as green staining. The Bowman's capsule (BC) and glomerular tuft (GT) areas are indicated by intermittent lines. For each glomerulus, both the BC and GT areas were measured: n=137 (WT) and n=111 (*Fuzzy*$^{-/-}$) glomeruli were analyzed. **B.** Distribution of Bowmen's capsule sizes plotted as a percentage of events for each 1000 µm^2. **C.** Statistical analysis of glomerular tuft area. **D.** Distribution of glomerular tuft area sizes as a percentage of events; each bin is 1000 µm^2. Scale bars are 25 µm.

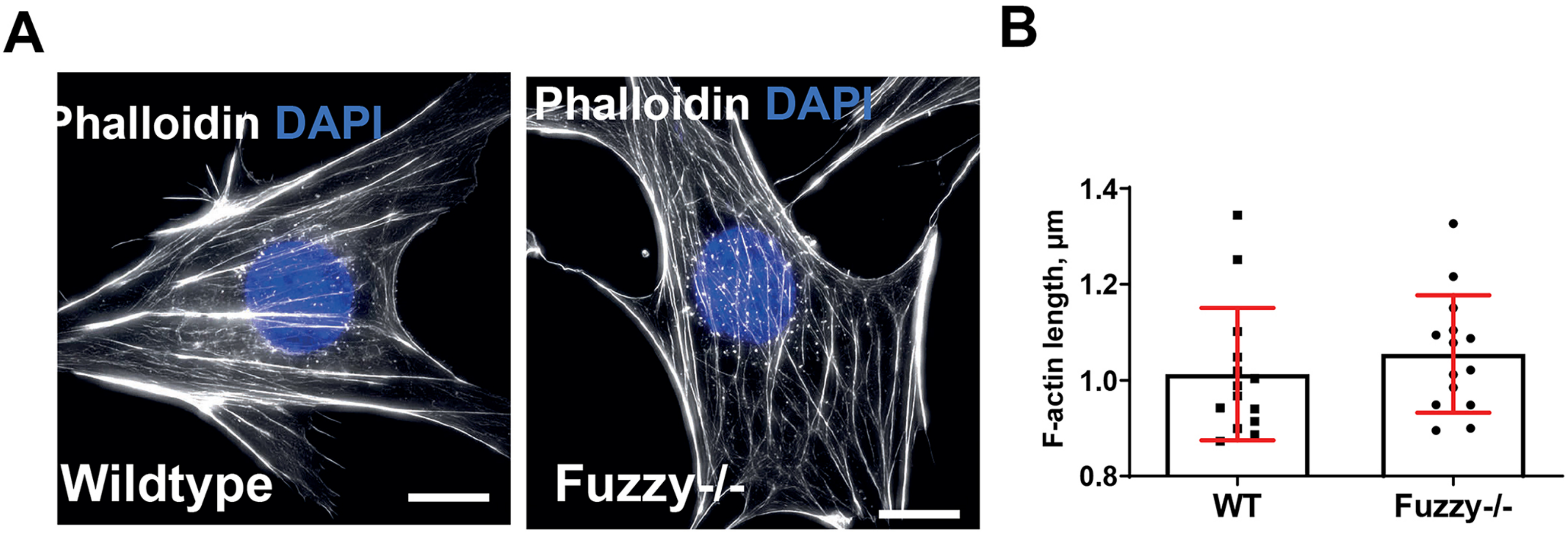

Fig. S3. Actin morphology in cultured wildtype and *Fuzzy*$^{-/-}$ MEFs. A. Representative Structural Illumination Microscopy images of wildtype and *Fuzzy*$^{-/-}$ MEFs stained with phalloidin to visualize F-actin stress fibers; scale bar 5 μm. **B.** Statistical analysis of F-actin fiber length. ImageJ was used to first define the threshold (thickness and length) that allowed to visualize F-actin strands traversing a cell. Then length of individual F-actin strands was measured, and an average F-actin stress fiber length for each cell was plotted. N= 14 (WT) and N=14 (*Fuzzy*$^{-/-}$) cells were analyzed. Note that there was no difference in the F-actin distribution between WT and *Fuzzy*$^{-/-}$ cells.

Development • Supplementary information

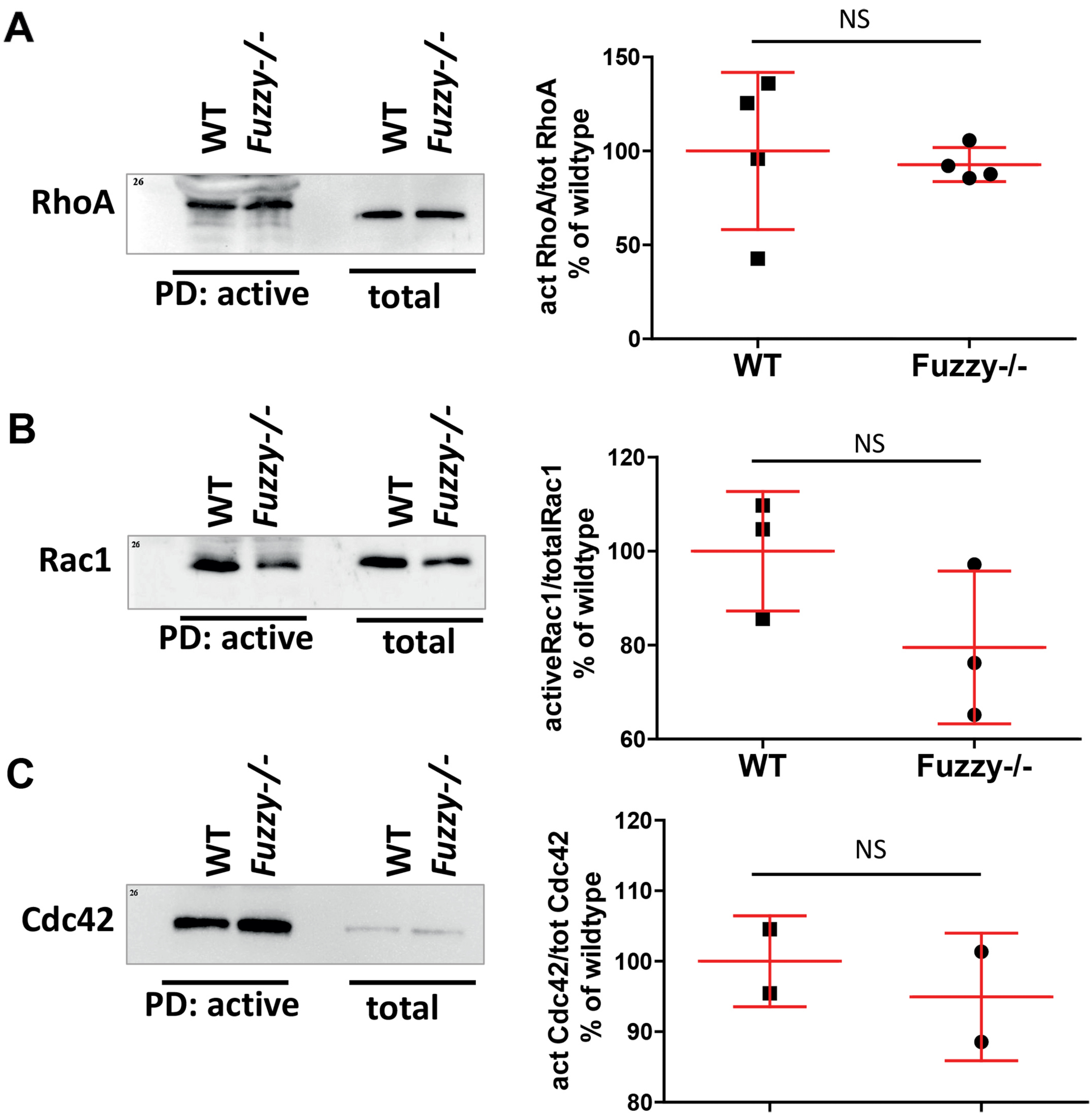

Fig. S4. Analysis of RhoA, CDC42 and Rac1 activity in WT and *Fuzzy-/-* MEFs by pull-down assays. A. RhoA pull-down assay in the whole cell lysates from wildtype and Fuzzy-/- MEFs. GST-Rhotekin construct (active RhoA interacting binding domain fused to GST, Addgene) was used to measure active RhoA as described previously (Benink and Bement, 2005) (left panel); RhoA was detected with anti-RhoA antibody (Cell signaling, 1:1000). Statistical analysis of 4 independent experiments is shown on the right panel. **B.** Rac1 pull-down assay in the whole cell lysates from wildtype and Fuzzy-/- MEFs. GST-CRIB-CDC42/Rac1 (active Cdc42/Rac1 interacting binding domain fused with GST) was used to measure Rac1 or Cdc2 activity as described in Matsuda et al. (2022); Rac1 was detected with anti-Rac1 antibody (Millipore, 1:1000, left panel). Statistical analysis of three independent experiments (right panel). **C.** Cdc42 pull-down assays in the whole cell lysates from wildtype and Fuzzy-/- MEFs; GST-CRIB-CDC42/Rac1 construct was used, Cdc42 was detected with anti-Cdc42 antibody (Santa Cruz, sc-87, 1:1000, left panel). Statistical analysis of two independent experiments (right panel).

Development • Supplementary information

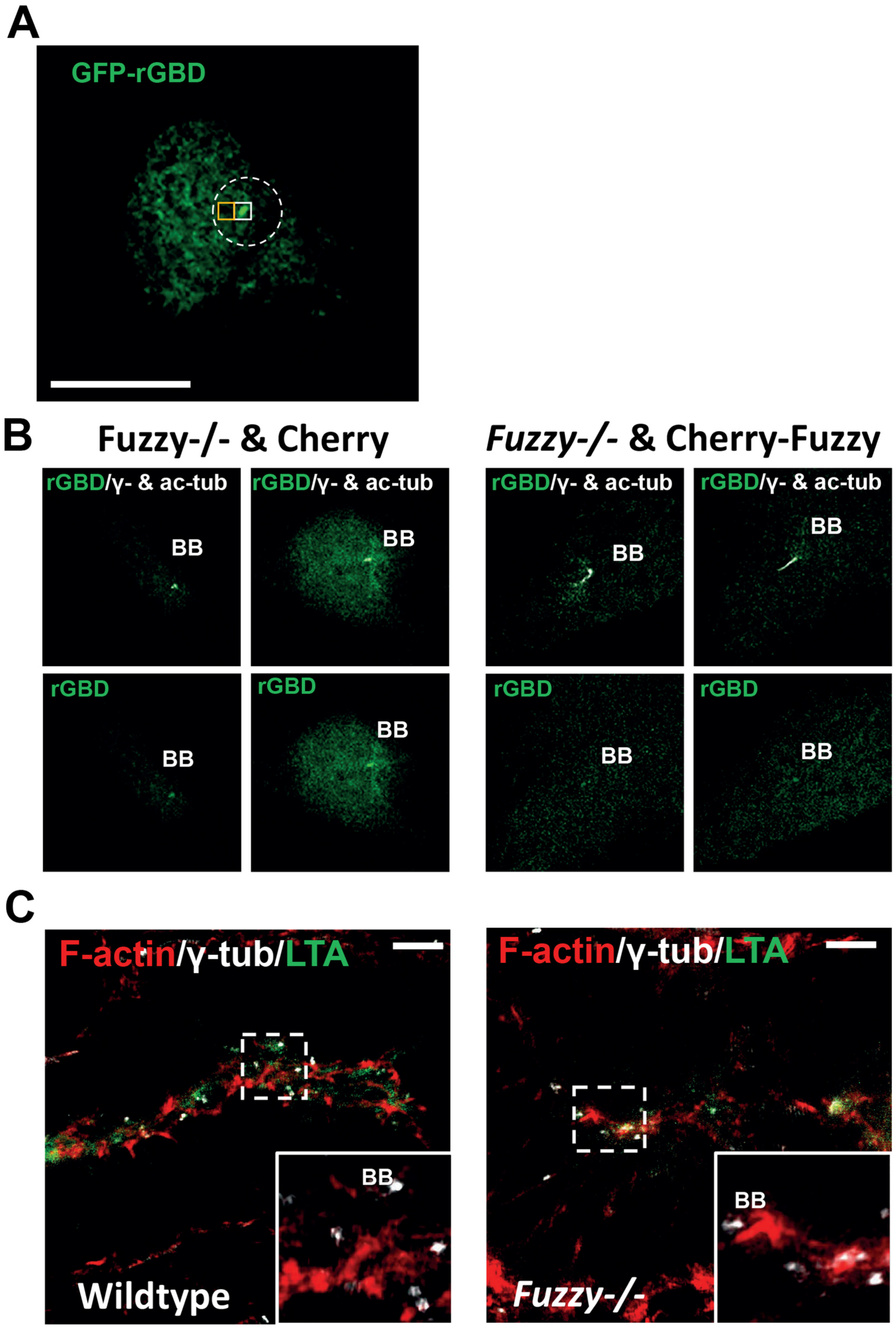

Development • Supplementary information

Fig. S5. RhoA activity and actin polymerization in wildtype and *Fuzzy$^{-/-}$* mutant cells. A. Measurement of the GFP-rGBD fluorescence intensity and its normalization for cytoplasmic GFP-eGBD expression. For each image of the GFP-rGBD-expressing cell, the position of the basal body was identified by immunostaining with anti-γ-tubulin antibody. Fluorescence intensity of the GFP-rGBD reporter within a 1.45μm^2 area containing basal body (white square) was measured. IF intensity in 3 different 1.45 μm^2 squares (orange square) within adjacent cytoplasm (circled area) was measured. The intensity of the rGBD (white square) was normalized for the average of the 3 measurements taken in the adjacent cytoplasm. scale bars 5μm. **B.** Additional images of rescue experiments (correspond to Fig. 4C). *Fuzzy$^{-/-}$* MEFs were co-transfected with GFP-rGBD and either Cherry or Cherry-Fuzzy. GFP-rGBD (green), anti-γ-tubulin and anti-acetylated tubulin antibodies detected a basal body and a rescued cilium (both white), respectively. The fluorescence intensity of the GFP-rGBD reporter was calculated as shown in image A. The presence of Cherry-Fuzzy reduces intensity of the GFP-rGBD at the basal body and rescues ciliogenesis. **C.** Additional images corresponding to Figure 4E. Proximal tubules were detected with LTA (green), F-actin (red) was detected with phalloidin and basal bodies (white) were visualized with anti-γ-tubulin antibody. Intensity of F-actin (red) at the basal body was measured in multiple images of proximal tubules. 3 embryos per genotype were used; scale bars 20μm, inlets are 2.5 x magnification of the small squares.

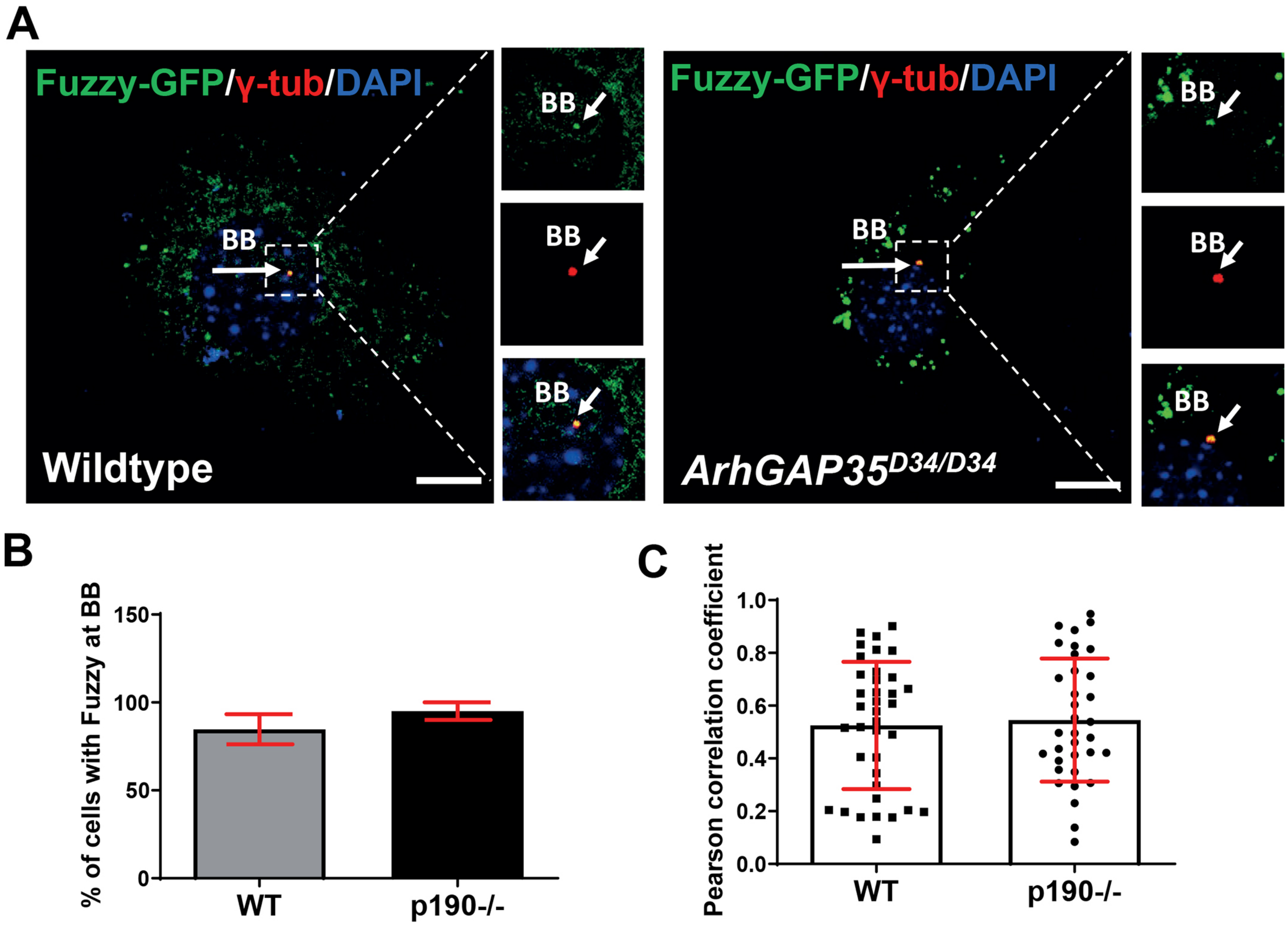

Fig. S6. GFP-Fuzzy localization at basal body in wildtype and *ArhGAP35*$^{D34/D34}$ MEFs. A. Confocal images of wildtype and *ArhGAP35*$^{D34/D34}$ MEFs expressing GFP-Fuzzy at basal body, visualized with anti-γ-tubulin antibody (red), scale bars 5 μm; inlets show separate channels at 2.5 time-magnification of the white box. Arrows point at the basal body, BB. **B.** Percentage of wildtype and *ArhGAP35*$^{D34/D34}$ MEFs with GFP-Fuzzy at the basal body. **C.** Pearson correlation coefficient between GFP-Fuzzy and basal body; n= 37 (WT) and n= 35 (*Fuzzy*$^{-/-}$) cells were analyzed in two independent experiments.

Development • Supplementary information

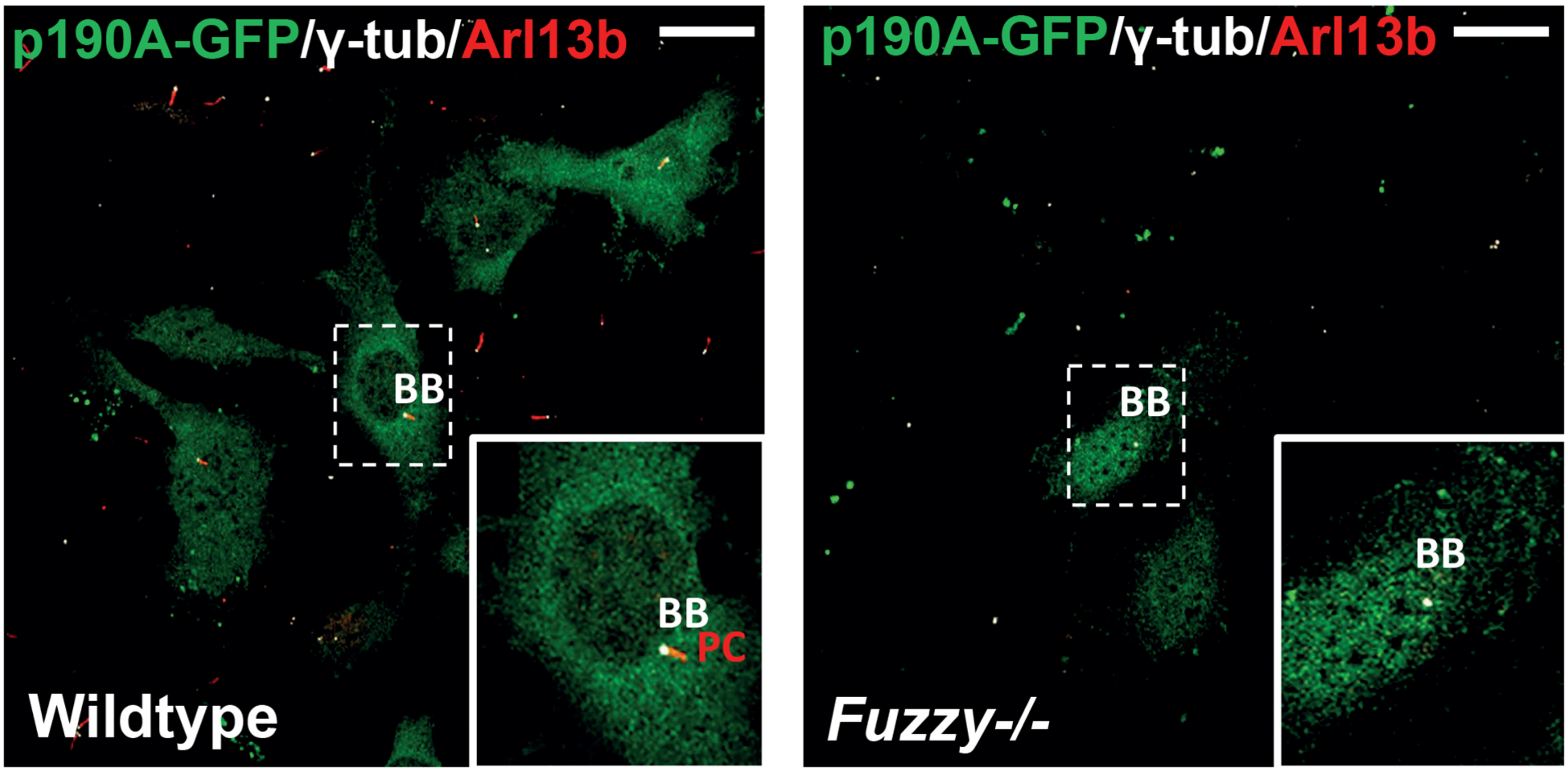

Fig. S7. Overexpression of p190A-GFP does not rescue ciliogenesis in *Fuzzy-/-* MEFs. Wildtype and Fuzzy-/- cells were transiently transfected with GFP-p190A and grown in the serum-free medium for 48 hours to induce ciliogenesis. Cilia were immunostained with anti-ARL13B antibody (red) and basal bodies -- with anti-γ-tubulin antibody (white). Transfected cells were visualized by green fluorescence. No cilia were detected in the Fuzzy-/- cells transiently overexpressing GFP-p190A, confirming our hypothesis that Fuzzy is required to recruit p190A to the basal body to keep actin polymerization in check. Scale bars 20 μm; inlets are 2.5 times magnified small squares in large images.

Development • Supplementary information

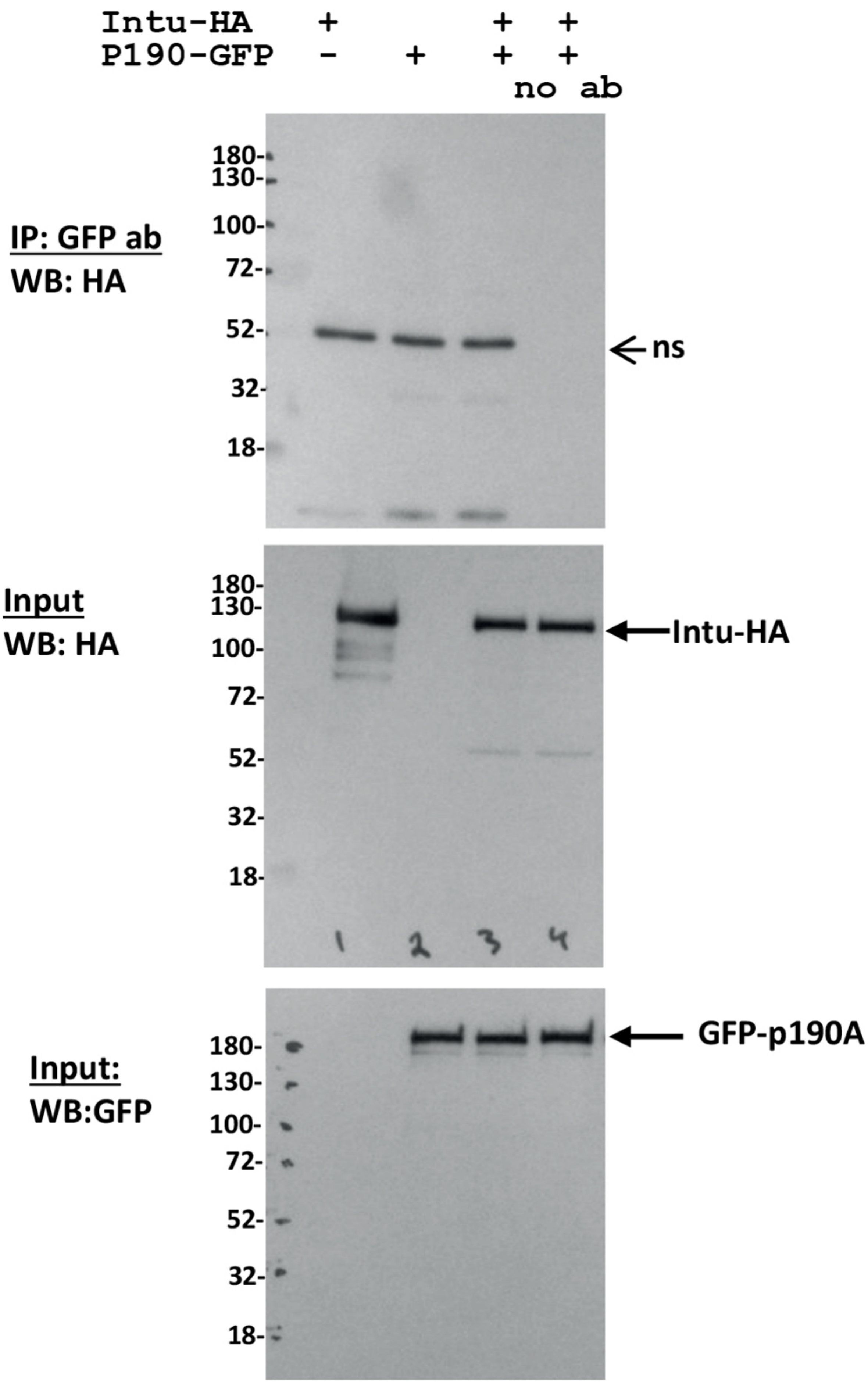

Fig. S8. p190A RhoGAP does not interact with CPLANE protein Inturned. Co-immunoprecipitation between human GFP-p190A and mouse Inturned-HA expressed in HEK293 cells. The top panel is co-IPs with anti-GFP antibody and detection with anti-HA-antibodies; two bottom panels are inputs to detect GFP-p190A and Inturned-HA with respective antibodies.

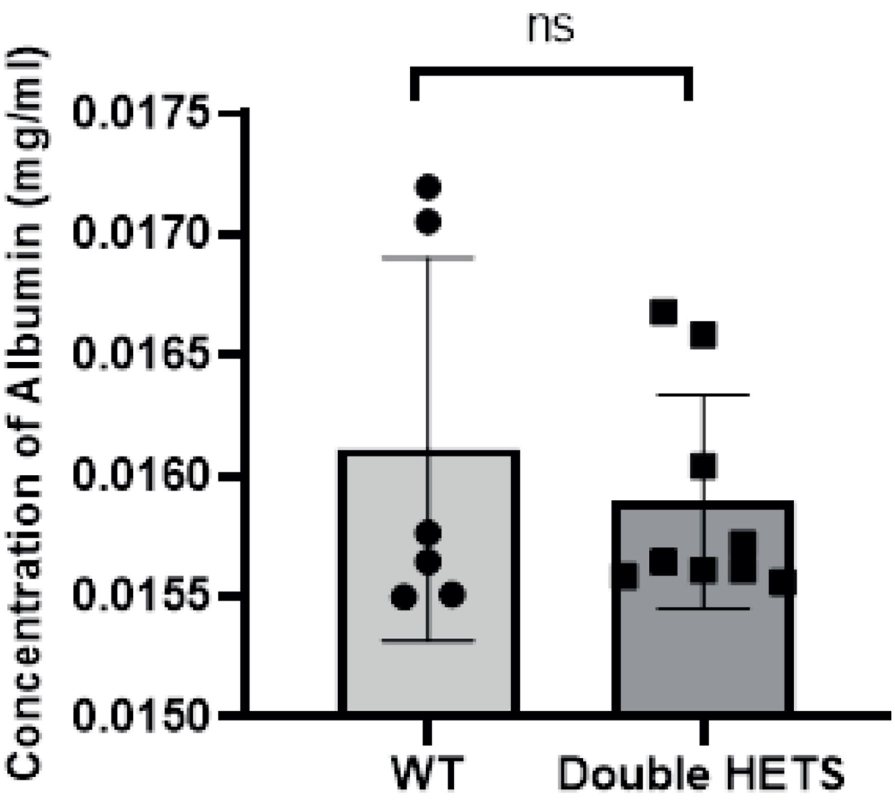

Fig. S9. Urine analysis of double heterozygous *Fuzzy*$^{+/-}$*;ArhGAP35*$^{+/D34}$ animals at 8 month. Urine was collected from n=6 (WT) and n=10 (*Fuzzy*$^{+/-}$*;ArhGAP35*$^{+/D34}$) 8 month-old animals and frozen at -80°C until use. All samples were analyzed at the same time with Mouse Albumin ELISA Assay kit (Bethyl labs, Texas, USA) following manufacturer recommendations. Standard deviations are shown. Student's unequal variance t-test was used to calculate the p-value.

Table S1. Genotype distribution in double heterozygous *Fuzzy*$^{+/-}$*;ArhGAP35*$^{+/D34}$ colony at weaning

Genotype	Percentage number of mice/Total number of mice*
WT	**30.0%** (86/295)
Fuzzy$^{+/-}$	**30.0%** (86/295)
ArhGAP35$^{+/D34}$	**22.0%** (65/295)
Fuzzy$^{+/-}$*;ArhGAP*$^{+/D34}$	**17.9%** (53/295)
Ratio of Male and Female in Double HETS	**54%:46%** (29:24)
P value for experimental vs expected genotype distribution (chi test)	**0.0069**

*All animals were genotyped at the time of weaning at 3 weeks of age. No pup death was detected prior to weaning. All adult animals appeared healthy and fertile.

Reference

Matsuda, J., Greenberg, D., Ibrahim, S., Maier, M., Aoudjit, L., Chapelle, J., Baldwin, C., He, Y., Lamarche-Vane, N., Takano, T. (2022). CdGAP maintains podocyte function and modulates focal adhesions in a Src kinase-dependent manner. *Sci Rep.* **12**, 18657. doi:10.1038/s41598-022-21634-1
