# Supplementary material for: The CPLANE protein Fuzzy regulates ciliogenesis by suppressing actin polymerization at the base of the primary cilium via p190A RhoGAP
Source: Development. 2024 Mar 28;151(6):dev202322. doi: 10.1242/dev.202322 (PMC11006408; doi:10.1242/dev.202322)
Supplement: Supplementary file 1 [file develop-151-202322_review_history.pdf]

The CPLANE protein Fuzzy regulates ciliogenesis by suppressing actin polymerization at the base of the primary cilium via p190A RhoGAP

Rhythm Sharma, Rita Kalot, Yossef Levin, Sima Babayeva, Nadezda Kachurina, Chen-Feng Chung, Karen J Liu, Maxime Bouchard and Elena Torban
DOI: 10.1242/dev.202322

Editor: Thomas Lecuit

Review timeline
Original submission: 4 September 2023
Editorial decision: 27 October 2023
First revision received: 19 December 2023
Accepted: 14 February 2024

Original submission

First decision letter

MS ID#: DEVELOP/2023/202322

MS TITLE: Ciliogenesis and Planar Cell Polarity Effector Fuzzy Controls Cilia Formation by Regulating Actin Polymerization at the Base of the Primary Cilium via p190A RhoGAP

AUTHORS: Rhythm Sharma, Rita Kalot, Yossef Levin, Sima Babayeva, Nadezda Kachurina, Chen-Feng Chung, Karen J Liu, Maxime Bouchard, and Elena Torban

I sincerely apologise for the long delay before coming back to you. I have now received all the referees reports on the above manuscript, and have reached a decision. The referees' comments are appended below, or you can access them online: please go to BenchPressand click on the 'Manuscripts with Decisions' queue in the Author Area.

The overall evaluation is positive and we would like to publish a revised manuscript in Development, provided that the referees' comments can be satisfactorily addressed. Please attend to all of the reviewers' comments in your revised manuscript and detail them in your point-by-point response. If you do not agree with any of their criticisms or suggestions explain clearly why this is so. If it would be helpful, you are welcome to contact us to discuss your revision in greater detail. Please send us a point-by-point response indicating your plans for addressing the referees' comments, and we will look over this and provide further guidance.

Reviewer 1

Advance summary and potential significance to field

In this manuscrit, the authors confirm that the protein Fuzzy controls ciliogenesis, leading to some pathologies, via p190A RhoGAP action on actin dynamic. While this was already known, they further demonstrate a rescue of the phenotype using ROCK inhibitors.

Comments for the author

© 2024. Published by The Company of Biologists under the terms of the Creative Commons Attribution License (https://creativecommons.org/licenses/by/4.0/).

The manuscrit is clear and the data support the claim. It can be improved notably regarding figures.

It is unclear how cilia length was measured from the Methods or pictures. This needs to be explained.
Line 128 typo on ciliogenesis.
Line 204: typo on Fuzzy Figure 2. The letter B should be shifted on the right with the graph. A higher magnification of the cilia would help appreciate qualitatively the length decrease.
Figure 3. An image of the controls in addition to the mutants would help appreciate the differences.
Figure 5. The figure can be improved, starting with the annotations' size and position, sometimes under the insets, sometimes on the edge of the photo. It is also difficult to appreciate the white channel (g-tubulin). In F, the size of the dots and squares can be reduced to match B and D.
Figure 6. Why is B not a distribution of data points like the other histograms?
In D, the illustration of the blots has to be improved, from the fonts, to the annotations.
Figure 11. The schematic could be clearer by adding a third panel for the rescue via fasudil.
The reference Hoffman HK, Prekeris R PMID: 35575063 doi: 10.1242/jcs.259030 should be cited.

<u>Reviewer 2</u>

Advance summary and potential significance to field

The current manuscript reports new ideas about how Fuzzy, a CPLANE protein involved in polarity and ciliogenesis, promotes ciliogenesis. The idea is that basal body-localized Fuzzy recruits p190AGAP which normally heads a pathway that inhibits actin polymerization. Too much actin polymerization in Fuzzy mutants is then proposed to be the cause of ciliogenesis defects, such that inhibitors of actin polymerization partially rescue ciliogenesis in Fuzzy mutant cells. Overall, this is a high quality manuscript with data ranging from immunofluorescence, actin drug treatments, and mouse genetic interaction work. The paper makes a novel advance and will be of interest to those trying to connect polarity/actin and ciliogenesis. I have two major comments about experiments and interpretations and a series of more minor issues that the authors should address, but overall I am supportive of publication.

Comments for the author

Major comments

The data in Figure 5 are not convincing. It is hard to "see" the result being reported in the images. The difference is evident in A but not C or E. Some suggestions for improvements: show BB counterstaining in part A; be more descriptive about how quantitation was performed (which part of the cytoplasm was used for normalization); can an additional basal body stain (e.g. a protein which localizes to basal bodies) be used for normalization (i.e. something which localizes to BB's but is unchanged across conditions); show more examples of images in Supplementary material.

The genetic interaction work between Fuzzy and ArhGAP35 is interesting and suggestive that these genes promote a similar pathway. Would cilia be expected to return in Fuzzy mutant MEFs transfected with ArhGAP35? The genetic interaction work hints that there is some overlapping/shared function but also that ArhGAP35 activity is not wholly dependent on Fuzzy because the phenotype gets worse when ArhGAP35 is lost in the Fuzzy mutant background. Thus, overexpression of ArhGAP35 might be enough to return ciliogenesis to Fuzzy mutants. This would be a more powerful demonstration of the two functioning in the same pathway than a more general "mixing/addition" of phenotypes in double mutants where the molecular reason for the mixing of phenotypes is hard to say.

Minor comments

The title is quite long-winded; the paper might have a bigger impact with a slightly punchier title.

© 2024. Published by The Company of Biologists under the terms of the Creative Commons Attribution License (https://creativecommons.org/licenses/by/4.0/).

Figures and parts of figures should be specifically referred to in the text, and be called upon approximately in order. Sometimes, this was not the case and it reduced the readability of the paper. For instance, in the first results section, broad statements are made and then "Figure 1" is called out, but it's not clear which part of Figure 1 to look at (the figure is divided into 5 panels, which are not mentioned specifically in the text).
Suggestion: be more precise about calling out specific parts of figures.

There are an unnecessarily large number of figures in this paper, currently 11 in total. Since several of these figures are broadly showing similar/overlapping things, I would combine several. For instance, the controls for the experiments in Figure 3 are in Figure 2, so these two can be combined. There are other instances where results are spread across figures which could be combined. Another example would be Figure 4, which is (broadly) showing the same thing as Figure 3 and so those could be combined. It would help make the paper more readable in general, with less jumping around. Suggestion: try to consolidate some of the figures to reduce the overall number and place relevant experimental results next to each other in the same figure.

Length is spelled wrong in many of the figures.

First revision

Author response to reviewers' comments

RESPONSE TO REVIEWERS:

We would like to thank the reviewers for their positive view of our initial submission and for constructive comments and suggestions. We addressed most of your comments and provide a detailed account of the revisions below.

Response to Reviewer 1

As requested by Reviewer 1, we added "Measurements of ciliary length" in the Materials and Methods.
As suggested, we merged Figure 2 and 3 (now revised Figure 2); it now includes side-by-side images of untreated Fuzzy-/- cells and the Fuzzy -/- cells treated with Y27632, Fasudil and Cytochalasin D. We hope that the message of the combined Figure 4 is now clearer.

We revised Figure 4 (former Figure 5) by adding inlets to panels A and C and by producing new images for panel E made on freshly stained sections of E14.5 wildtype and mutant embryos. In addition, we now provide a new Supplemental Figure 5 where we explain how GFP-rGBD fluorescence intensity was measured (Fig. S5 A). In this new Supplemental Figure 5, we also included additional images for Figure 4 panels C and E. The graph in Figure 4, panel F was resized, as recommended.

Figure 5 (former Figure 6), panel B: in this panel, we calculated the percentage of the wildtype and Fuzzy-/- mutant cells with or without GFP-p190A at the basal body. In each experiment, we scored multiple cells with a binary answer of "yes" or "no". We repeated this experiment 3 times, and thus, could calculate standard deviations. If we used scattered dot plots, we would then be able to show only 3 dots (average percentage of the cells with "Yes" GFP-p190A expression at the basal body for each of the 3 experiments); we think this would somewhat misrepresent our data. For panel D, we resized the fonts, realigned and numbered all lanes for all western blot panels.

Figure 10 (former Figure 11) – we reworked the schematic, as suggested, to add a third panel showing that despite the absence of Fuzzy or p190A at the basal body, ciliogenesis could be rescued with ROCK or actin polymerization inhibitors.

© 2024. Published by The Company of Biologists under the terms of the Creative Commons Attribution License (https://creativecommons.org/licenses/by/4.0/).

We carefully checked the manuscript for spelling mistakes and corrected the errors we found. All textual changes were made with the TrackChanges function ON and can be seen in the “red ink” copy of the revised manuscript.

Response to Reviewer 2:

Reviewer 2 commented that the data in Figure 5 (revised Figure 4) were not convincing. We reworked this figure according to the Reviewer’s advice. Panel A: we added inlets with the staining for the basal body (white, visualized with gamma-tubulin antibody). We also added more details in the Material and Method section on how we measured cytoplasmic GFP-rGBD intensity to allow normalization for transfection efficiency in each analyzed cell and depicted this process in the new Supplemental Figure 5, panel A. For panel C, we added an inlet to show basal body and primary cilium immunodetected with gamma-tubulin and acetylated-tubulin antibodies; cilium could be detected only in the cells transfected with Cherry-Fuzzy but not with Cherry-empty expression vectors. Please note that BB and the cilium are visualized in the same channel (white) since we used blue (DAPI) to visualize nuclei, red (to visualize transfected cells by Cherry expression, not shown), and green (to visualize GFP-rGBD), thus, exhausting 4-channel capacity of our confocal microscope. Panel E: We repeated the experiment on fresh E14.5 wildtype and Fuzzy-/- frozen sections and now present new images. For Figure 4 panels C and E, we now provide new Supplemental Figures S5, panels B and C, respectively, that show additional images. We hope that the reworked Figure 4 (former5) and new supplemental Figure S5 convince the reviewers and the readers of the accuracy of the reported results.

Reviewer2 suggested that “.. overexpression of ArhGAp35 might be enough to return ciliogenesis to Fuzzy mutant. This would be a more powerful demonstration of the two functioning in the same pathway that a more general “mixing/addition” of phenotypes in double mutants…”.
Our hypothesis posits that Fuzzy recruits p190A to the basal body where p190A controls actin polymerization favoring ciliogenesis, however, when Fuzzy is lost, p190A does not localize to the basal body. The loss of p190A at the basal body leads to increased RhoA activity and decreased ciliation. Indeed, Figure 5 (previously Figure 6) shows that in the absence of Fuzzy, p190A does not localize to the basal body. We now provide an additional Supplemental Figure 7 showing that Fuzzy-/- cells transfected with GFP-p190A do not generate primary cilium. From these experiments, we conclude that without Fuzzy, p190A does not reach the basal body and thus, ciliogenesis cannot be rescued by overexpressing p190A alone. We now discuss this point in both the Results and the Discussion sections.

We have referred to the specific parts of the Figures in the Results. We hope that this facilitates reading.

As recommended, we merged Figures 2 and 3 into a revised Figure 2. We choose to keep Figure 4 separately to avoid crowding in Figure 2 – although we do agree that the ideas in the original Figures 2, 3 and 4 are similar. We mentioned Figures and specific panels in the text of the Results section.

We carefully checked the paper for spelling mistakes and corrected the ones we found. All changes in the text are reflected in the “red ink” copy that shows all modification with the TrackChanges function ON.

We trust that our revisions have answered your concerns and improved our manuscript to your satisfaction. We hope that now our manuscript meets the stringent criteria of “Development” for publication.

Sincerely,
Elena Torban

Second decision letter

© 2024. Published by The Company of Biologists under the terms of the Creative Commons Attribution License (https://creativecommons.org/licenses/by/4.0/).

MS ID#: DEVELOP/2023/202322

MS TITLE: CPLANE Protein Fuzzy Regulates Ciliogenesis by Suppressing Actin Polymerization at the Base of the Primary Cilium via p190A RhoGAP

AUTHORS: Rhythm Sharma, Rita Kalot, Yossef Levin, Sima Babayeva, Nadezda Kachurina, Chen-Feng Chung, Karen J Liu, Maxime Bouchard, and Elena Torban
ARTICLE TYPE: Research Article

I apologise for the delay in getting back to you. But I am happy to tell you that your manuscript has been accepted for publication in Development, pending our standard ethics checks.

Reviewer 2

Advance summary and potential significance to field

The current manuscript reports new ideas about how Fuzzy, a CPLANE protein involved in polarity and ciliogenesis, promotes ciliogenesis. The idea is that basal body-localized Fuzzy recruits p190AGAP which normally heads a pathway that inhibits actin polymerization. Too much actin polymerization in Fuzzy mutants is then proposed to be the cause of ciliogenesis defects, such that inhibitors of actin polymerization partially rescue ciliogenesis in Fuzzy mutant cells. Overall, this is a high quality manuscript with data ranging from immunofluorescence, actin drug treatments, and mouse genetic interaction work. The paper makes a novel advance and will be of interest to those trying to connect polarity/actin and ciliogenesis. During the previous round of review, I had two major comments and both have been well addressed in this new submission.

Comments for the author

I still personally believe the title could be improved, but overall I am highly supportive of publication of this interesting manuscript in Development.

© 2024. Published by The Company of Biologists under the terms of the Creative Commons Attribution License (https://creativecommons.org/licenses/by/4.0/).
